# RNF141 interacts with KRAS to promote colorectal cancer progression

**DOI:** 10.1038/s41388-021-01877-4

**Published:** 2021-08-03

**Authors:** Jiuna Zhang, Xiaoyu Jiang, Jie Yin, Shiying Dou, Xiaoli Xie, Ting Liu, Yijun Wang, Shuling Wang, Xue Zhou, Dongxuan Zhang, Huiqing Jiang

**Affiliations:** 1 Department of Gastroenterology, The Second Hospital of Hebei Medical University, Hebei Key Laboratory of Gastroenterology, Hebei Institute of Gastroenterology, Shijiazhuang, P. R. China; 2 Department of Gastroenterology, The Affiliated Hospital of Hebei Engineering University, Handan, P. R. China

**Keywords:** Gene therapy, Colorectal cancer, Cancer genetics

## Abstract

RING finger proteins (RNFs) play a critical role in cancer initiation and progression. RNF141 is a member of RNFs family; however, its clinical significance, roles, and mechanism in colorectal cancer (CRC) remain poorly understood. Here, we examined the expression of RNF141 in 64 pairs of CRC and adjacent normal tissues by real-time PCR, Western blot, and immunohistochemical analysis. We found that there was more expression of RNF141 in CRC tissue compared with its adjacent normal tissue and high RNF141 expression associated with T stage. In vivo and in vitro functional experiments were conducted and revealed the oncogenic role of RNF141 in CRC. RNF141 knockdown suppressed proliferation, arrested the cell cycle in the G1 phase, inhibited migration, invasion and HUVEC tube formation but promoted apoptosis, whereas RNF141 overexpression exerted the opposite effects in CRC cells. The subcutaneous xenograft models showed that RNF141 knockdown reduced tumor growth, but its overexpression promoted tumor growth. Mechanistically, liquid chromatography-tandem mass spectrometry indicated RNF141 interacted with KRAS, which was confirmed by Co-immunoprecipitation, Immunofluorescence assay. Further analysis with bimolecular fluorescence complementation (BiFC) and Glutathione-S-transferase (GST) pull-down assays showed that RNF141 could directly bind to KRAS. Importantly, the upregulation of RNF141 increased GTP-bound KRAS, but its knockdown resulted in a reduction accordingly. Next, we demonstrated that RNF141 induced KRAS activation via increasing its enrichment on the plasma membrane not altering total KRAS expression, which was facilitated by the interaction with LYPLA1. Moreover, KRAS silencing partially abolished the effect of RNF141 on cell proliferation and apoptosis. In addition, our findings presented that RNF141 functioned as an oncogene by upregulating KRAS activity in a manner of promoting KRAS enrichment on the plasma membrane in CRC.

## Introduction

Colorectal cancer ranks as the fourth most deadly cancer in the world, resulting in approximately 900,000 deaths annually, and accounts for almost 10% of all annually diagnosed cancers globally [1, 2]. Pathogenetic mechanisms of CRC were mainly attributable to an accumulation of distinct genetic and epigenetic alterations [3–6]. Interestingly, more than 40% of colorectal cancers harbor KRAS mutation [3, 5, 7], resulting in the continuous activation of its downstream Raf–MEK-ERK signaling pathway and malignant transformation [8]. EGFR targeted therapy drugs, such as cetuximab and panitumumab, are effective as first-line therapy in RAS and BRAF wild-type metastatic colorectal cancer (mCRC). However, these medications have limitations in mCRC patients with KRAS mutation [9, 10] and there are no effective anti-RAS therapies [11, 12]. Thus, it is crucial to identify new biomarkers or targets for diagnosing and treating CRC.

The RING (Really Interesting New Gene) finger (RNF) proteins, owing to RING finger domains based on structural similitude, are involved in a variety of biological processes [13, 14]. Its dysregulation contributed to multiple human disorders, particularly malignancy [15–17]. Over 600 RNF domain proteins have been identified [13], and increasing studies have shown that these proteins regulate either tumor-suppressive or tumor-promotive pathways, indicating that they could suppress or accelerate carcinogenesis depending on the nature of their targets [18, 19]. For example, RNF43/ZNRF3, a negative regulator of Wnt signaling, recognizes and mediates the ubiquitylation of specific Wnt frizzled receptors, which subsequently undergo endocytosis and degradation, leading to suppressed Wnt cascade and tumor suppression [20, 21]. On the other hand, RNF183 exerts a positive effect on the proliferation and metastasis of CRC cells by activating NF-κB-IL-8 axis [22]. Importantly, the previous studies find that RNF141 promotes spermatogenic cell proliferation and sperm maturation, as well as motility and fertilization in mouse spermatogenesis [23], and exerts a broad function on the development of embryos [24]. Another study shows that there is an increased expression of RNF141 in Barrett’s esophagus or esophageal adenocarcinoma than that in normal esophagus [16]. However, its clinical significance, roles, and mechanism in CRC remain poorly understood.

Here, we report for the first time that RNF141 was upregulated in CRC tissues, and was positively correlated with T stage. Moreover, both in vivo and in vitro data showed that RNF141 induced CRC cell proliferation, migration, invasion, and metastasis, but inhibited apoptosis through increasing the level of activated KRAS. These findings strongly indicated that RNF141 promoted CRC tumorigenesis and was a potential therapeutic target.

## Results

### RNF141 is upregulated in CRC tissues

**Fig. 1:**
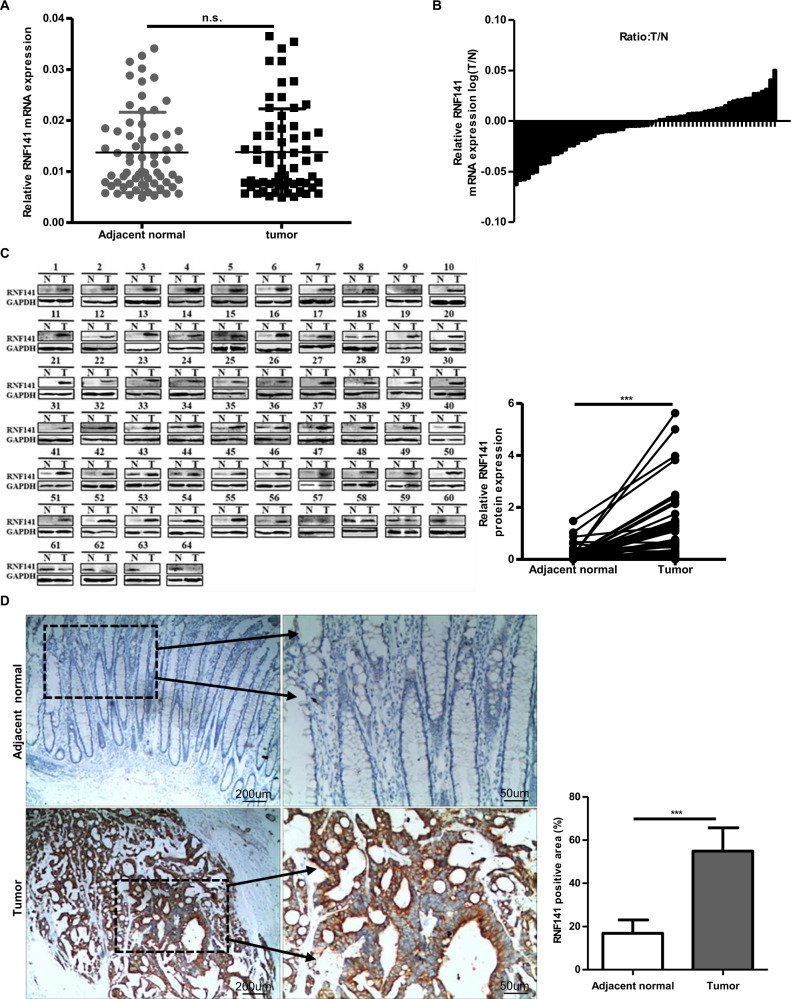
RNF141 was upregulated in CRC tissues. **A** The expression of RNF141 mRNA was verified by RT-PCR in 64 pairs of CRC and adjacent normal tissues. **B** The ration (T/N) of RNF141 mRNA was shown as log (T/N). **C** The RNF141 protein levels in 64 pairs of CRC and adjacent normal tissues was analyzed by Western blot. **D** RNF141 expression was analyzed by IHC using RNF141 antibody in CRC tissue, and typical photos were presented (scale bar, 200 μm; inset scale bar, 50 μm). Adjacent normal was the adjacent normal colorectal tissue from the same case. GAPDH glyceraldehyde-3-phosphate dehydrogenase. ***P < 0.001. T tumor, N adjacent normal tissues.

To identify whether RNF141 was involved in colorectal tumorigenesis, we first examined the mRNA and protein levels of RNF141 in 64 paired CRC and corresponding adjacent normal tissues. There was no significant difference in RNF141 mRNA level between CRC and peritumor tissues (Fig. 1A, B, *P* = 0.625). However, both Western blot and IHC analyses showed that, compared with adjacent normal tissues, RNF141 protein level was significantly elevated in CRC tissues (Fig. 1C, D). Additionally, IHC studies revealed that RNF1141 protein was mainly distributed in the membrane and cytoplasm of tumor cells. Next, we investigated the association between RNF141 protein expression with patients’ clinicopathological characteristics. As shown in Table 1, RNF141 expression did not correlate with gender, age, tumor location, the differentiation degree of tumors, lymph node metastasis, or AJCC stage but correlated with T stage, with a higher expression found in higher T stage (*P* = 0.019). Together, these results indicated that RNF141 upregulation might play an important role in colorectal tumorigenesis.

**Table 1:** Correlation between RNF141 expression level and clinicopathological characteristics in 64 CRC patients.

Variables	No. of patients *N* (%)	RNF141 expression (${\bar{\boldsymbol x}}$ ± *s*)	*P* value
**All cases**	64 (100%)		
**Gender**			0.189
Male	34 (53.13%)	1.02 ± 2.07	
Female	30 (46.87%)	0.67 ± 0.29	
**Age**			0.143
≥60	38 (59.38%)	1.03 ± 1.39	
<60	26 (40.62%)	0.61 ± 0.98	
**Tumor location**			0.703
Colon	30 (46.87%)	0.91 ± 0.96	
Rectum	34 (53.13%)	0.81 ± 1.54)	
**Differentiation**			0.879
Well or moderately	52 (81.25%)	0.84 ± 1.12)	
Poorly	12 (18.75%)	0.90 ± 2.06	
**T stage**			0.019(*)
T1 or T2	14 (21.88%)	0.46 ± 0.21	
T3 or T4	50 (78.12%)	0.97 ± 1.49	
**Lymph node metastasis**			0.675
No	40 (62.50%)	0.81 ± 1.50	
Yes	24 (37.50%)	0.93 ± 0.88	
**AJCC stage**			0. 172
I or II	39 (60.94%)	0.70 ± 1.06	
III or IV	25 (39.06%)	1.10 ± 1.51	

AJCC American Joint Committee on Cancer, T tumor; *P < 0.05.

### RNF141 promotes cell proliferation and facilitates the G1/S transition in vitro

**Fig. 2:**
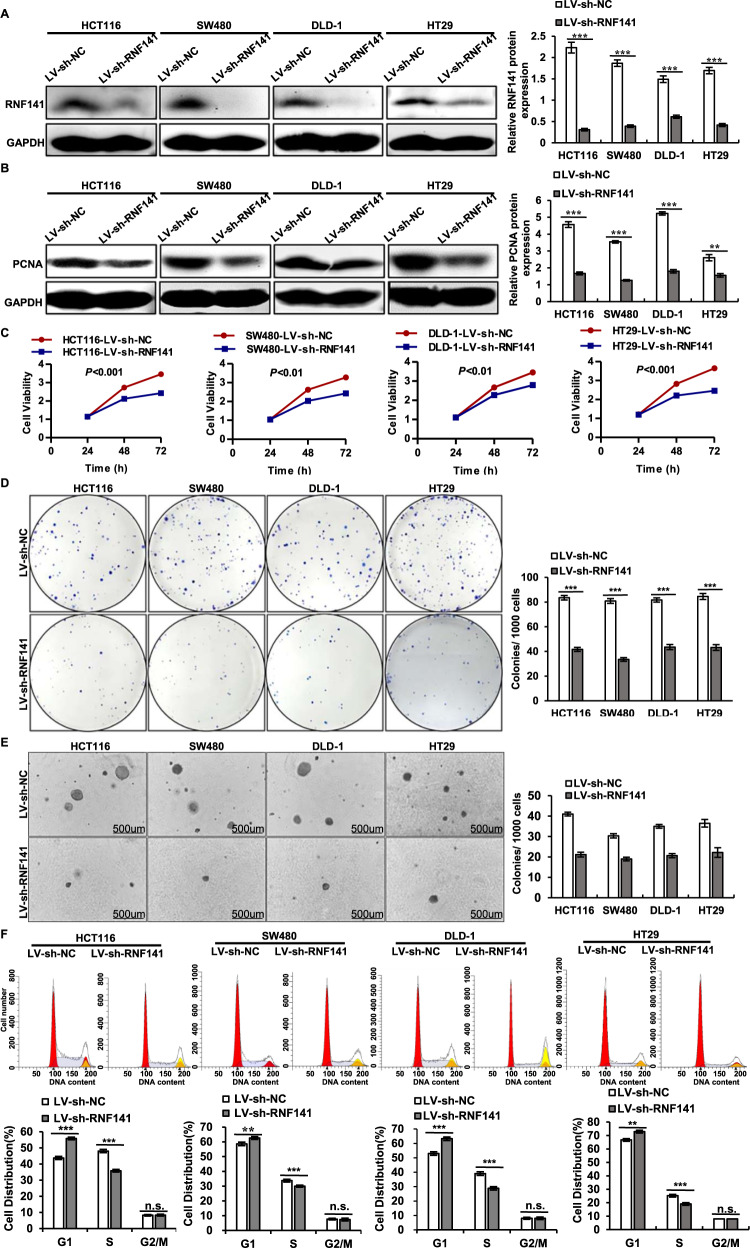
The silence of RNF141 suppresses cell proliferation and inhibits G1/S transition in vitro. **A**, **B** The silence efficiency of RNF141 was confirmed (**A**) and effects of RNF141 silence on PCNA expression in CRC cells was detected by Western blot (**B**). **C**–**E** Cell proliferation were further verified by CCK-8 assays (**C**), plate colony formation assay (**D**), and soft agar assay (**E**). Scale bar, 500 μm. **F** Flow cytometry results exhibited the cell phase distribution. Graph bars showed means ± SD from at least three independent experiments, **P < 0.01, ***P < 0.001 vs indicated group. *n.s*. indicates no significance.

To further investigate the role of RNF141 in CRC cell proliferation, RNF141 expression was silenced by LV-sh-RNF141 transfection and RNF141 overexpression was induced through LV-RNF141 transfection. The transfection efficiency of RNF141 was confirmed by Western blot (Fig. 2A for RNF141 knockdown, Fig. s1A for RNF141 overexpression). As a result, RNF141 knockdown led to a decrease in proliferating cell nuclear antigen expression (PCNA) (Fig. 2B), while RNF141 overexpression induced an increase in PCNA expression in HCT116, SW480, and DLD-1 cells (Fig. s1B). Moreover, CCK8 assays revealed that RNF141 knockdown markedly attenuated the cell viability (Fig. 2C), whereas RNF141 overexpression exhibited opposite effect (Fig. s1C). Simultaneously, both plate colony formation assay and soft agar colony formation assay demonstrated that RNF141 knockdown significantly decreased colony formation rate in CRC cells (Fig. 2D, E). However, in comparison with CRC cells transfected with LV-NC, an increase in size and number of colonies was observed in LV-RNF141-transfected CRC cells (Fig. s1D, E), which further verified the promotive effect of RNF141 on cell proliferation.

To explore the mechanism by which RNF141 accelerated cell proliferation, we examined the effect of RNF141 on cell cycle progression by flow cytometry. As presented in Fig. 2F, compared with LV-sh-NC group, LV-sh-RNF141 group all showed a sharp increased percentage of cells in G1 phase (55.88 ± 0.84% vs 43.73 ± 0.97%; 62.61 ± 0.96% vs 58.60 ± 1.17%; 63.21 ± 1.22% vs 52.99 ± 1.23%; 72.91 ± 1.01% vs 66.76 ± 0.96%), and a corresponding reduction of proportion in S phase (35.81 ± 0.82% vs 48.03 ± 1.01%; 29.98 ± 0.65% vs 33.73 ± 0.95%; 28.79 ± 1.22% vs 39.01 ± 1.23%; 19.08 ± 11.02% vs 25.24 ± 0.96%). There was no difference in G2/M phase between CRC cells transfected with LV-sh-RNF141 and their corresponding control cells. On the contrary, RNF141 overexpression induced G1/S transition resulting in a decreased percentage of cells in G1 phase (37.77 ± 0.69% vs 44.70 ± 1.03%; 51.48 ± 0.56% vs 59.16 ± 0.62%; 43.15 ± 0.92% vs 51.71 ± 1.20%; 55.39 ± 0.90% vs 66.24 ± 1.24%), and a promoted percentage in S phase (54.18 ± 0.70% vs 47.12 ± 0.97%; 41.31 ± 1.18% vs 33.40 ± 0.90%; 48.85 ± 0.92% vs 40.46 ± 1.29%; 36.61 ± 0.90% vs 25.76 ± 1.294%). In addition, RNF141 overexpression was not yet found to affect G2/M phase (Fig. s1F). These results suggested that RNF141 accelerated CRC cell cycle progression by facilitating the G1/S transition to promote cell proliferation.

### RNF141 inhibits cell apoptosis

**Fig. 3:**
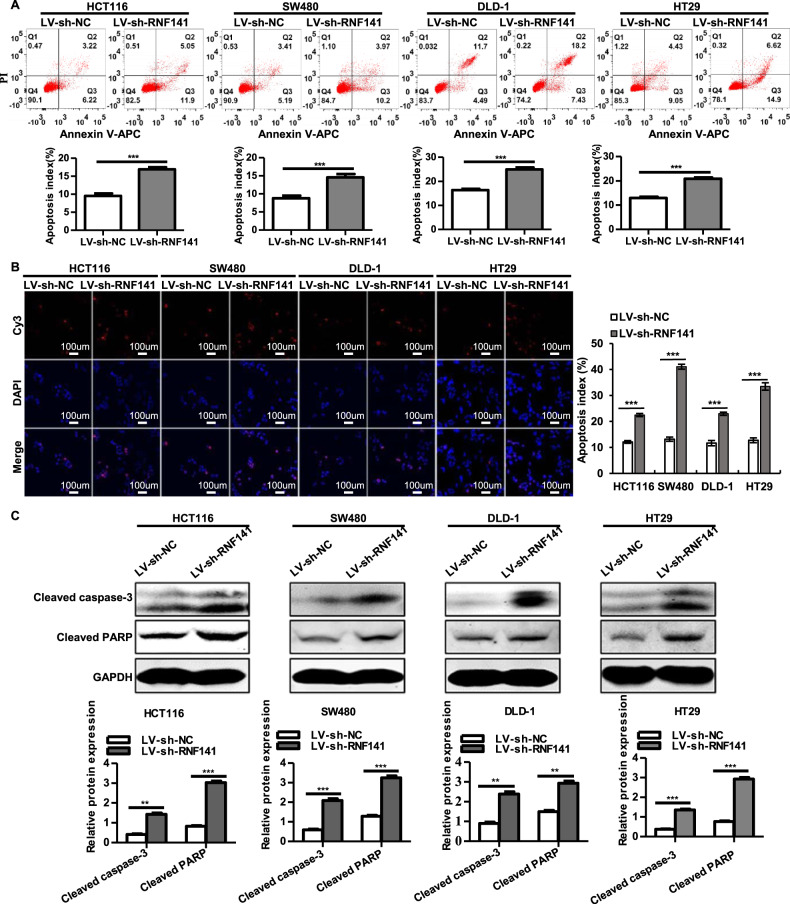
Knockdown of RNF141 promotes cell apoptosis. **A**, **B** Cell apoptosis was determined by Annexin V-APC/PI staining (**A**) and TUNEL technology, Scale bar, 100 μm (**B**). **C** Activated apoptosis-related proteins cleaved caspase-3 and PARP were assessed by Western blot. All values were the mean ± S.D. from three independent experiments, **P < 0.01, ***P < 0.001.

We next analyzed whether RNF141 exerted any impacts on cell apoptosis in colorectal tumorigenesis. When compared to their corresponding control cells, LV-sh-RNF141-transfected CRC cells exhibited a higher percentage of apoptotic cells (Annexin V-positive cells) in HCT116 (16.92 ± 0.37% vs 9.56 ± 0.43%), SW480 (14.55 ± 0.85% vs 8.80 ± 0.52%), DLD-1 cells (24.95 ± 0.66% vs 16.37 ± 0.27%) and HT29 (20.87 ± 0.54% vs 12.93 ± 0.51%) (Fig. 3A). In addition, compared with LV-NC group, a significant diminution of Annexin V-positive cells was observed in RNF141 overexpressing HCT116 (2.8 ± 0.13% vs 9.04 ± 0.27%), SW480 (3.82 ± 0.55% vs 9.41 ± 0.42%), DLD-1 cells (1.30 ± 0.07% vs 9.00 ± 0.33%) and HT29 (7.43 ± 0.36% vs 16.17 ± 0.61%) (Fig. s2A). Meanwhile, TUNEL assay revealed similar data that RNF141 knockdown contributed to an increase of TUNEL-positive cells in HCT116 (22.47 ± 0.61% vs 12.12 ± 0.61%), SW480 (41.07 ± 0.96% vs 13.17 ± 0.82%), DLD-1 cells (22.97 ± 0.61% vs 11.65 ± 1.03%) and HT29 cells (33.45 ± 1.42% vs 12.75 ± 0.94%) compared with their corresponding control groups, respectively (Fig. 3B). In contrast, RNF141 overexpression remarkedly diminished TUNEL-positive cells in HCT116 (2.73 ± 0.38% vs 12.03 ± 0.28%), SW480 (3.42 ± 0.29% vs 10.75 ± 0.33%), DLD-1 cells (2.75 ± 0.187% vs 13.65 ± 0.31%) and HT29 cells (3.58 ± 0.37% vs 11.72 ± 0.79%) (Fig. s2B). To further validate this, we examined whether altered RNF141 expression affected active forms of apoptosis-related proteins’ expression, including cleaved-caspase-3 and cleaved-PARP. The results were in line with expectation that silence of RNF141 significantly upregulated the expressions of cleaved-caspase-3 and cleaved-PARP in HCT116, SW480, DLD-1, and HT29 cells (Fig. 3C), and as presented in Fig. s2C, enhanced RNF141 displayed suppressive effects on cleaved caspase-3 and cleaved caspase-3 expression. Taken together, these data indicated that RNF141 inhibited cell apoptosis.

### RNF141 accelerates cell migration and invasion and facilitates the HUVEC tube formation in vitro

**Fig. 4:**
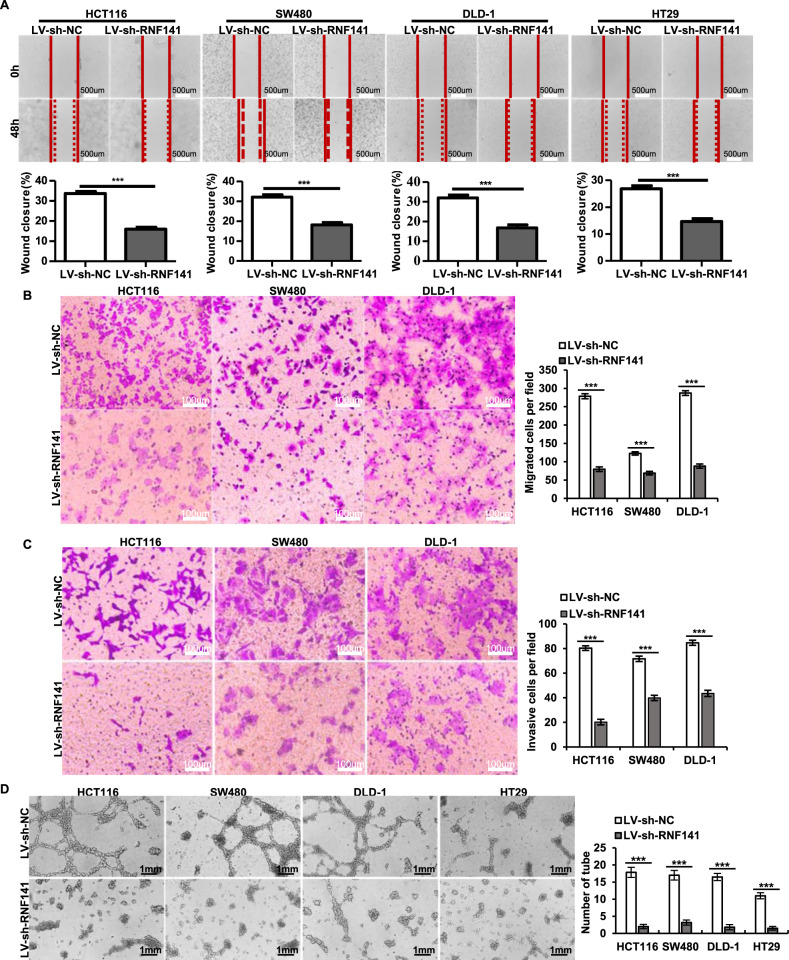
Silencing of RNF141 impairs cell migration and invasion and diminishes the HUVEC tube formation in vitro. **A** Wound-healing assay was performed to evaluate the effect of RNF141 overexpression on cell migration at 48 h after scratching (0 h). The percentage of wound closure was calculated as: (area of original wound-area of actual wound) / area of original wound × 100%. Scale bar, 500 μm. **B**, **C** Representative photos of the transwell assay showed the migrated and invasive cells of HCT116, SW480, DLD-1, and HT29 transfected with indicated lentivirus. Scale bar, 100 μm. **D** Tube formation assay was conducted in vitro using HUVEC cells. LV-sh-NC or LV-sh-RNF141 transfected HCT116, SW480, DLD-1, and HT29 cells were cultured in serum-free media for 24 h, and then conditioned media were collected. HUVEC cells were incubated in conditioned media for 12 h and then tube formation was photoed. Scale bar, 1 mm. Data represented the mean ± S.D. from three independent experiments, ***P < 0.001 vs indicated groups.

Subsequently, wound healing assay and transwell assay were employed to evaluate the effect of RNF141 on cell migration and invasion. RNF141 knockdown significantly impaired the migratory capacity of HCT116, SW480, DLD-1, and HT29 cells, resulting in an evident decrease of wound closure (Fig. 4A). Similar to the results of wound healing assay, the results of the migration transwell assay showed that the numbers of migrated CRC cells in LV-sh-RNF141 group were significantly less than those in LV-sh-NC group (Fig. 4B). Consistent with migration results, it was validated via the invasion transwell assay that CRC cell’s invasive ability was notably reduced by RNF141 knockdown (Fig. 4C). To confirm the above findings, migration and invasion assays were also conducted in CRC cells with RNF141 overexpressing, and the results showed that RNF141 overexpression exerted promotive effects on cell migration and invasion that LV-RNF141 group showed a significant increase of wound closure compared to LV-NC group (Fig. s3A). The number of migrated CRC cells (Fig. s3B) and the number of invasive CRC cells (Fig. s3C) was increased in the LV-RNF141 group compared to those in the LV-NC group. These data demonstrated that RNF141 accelerated cell migration and invasion.

Meanwhile, to determine whether RNF141 promoted angiogenesis, we carried out the tube formation assay in vitro. As shown in Fig. 4D, silence of RNF141 resulted in disruption of tube formation. Conversely, overexpression of RNF141 induced a positive influence on tube formation (Fig. s3D), suggesting that RNF141 facilitated the tube formation.

### RNF141 promotes cell tumorigenicity in vivo

**Fig. 5:**
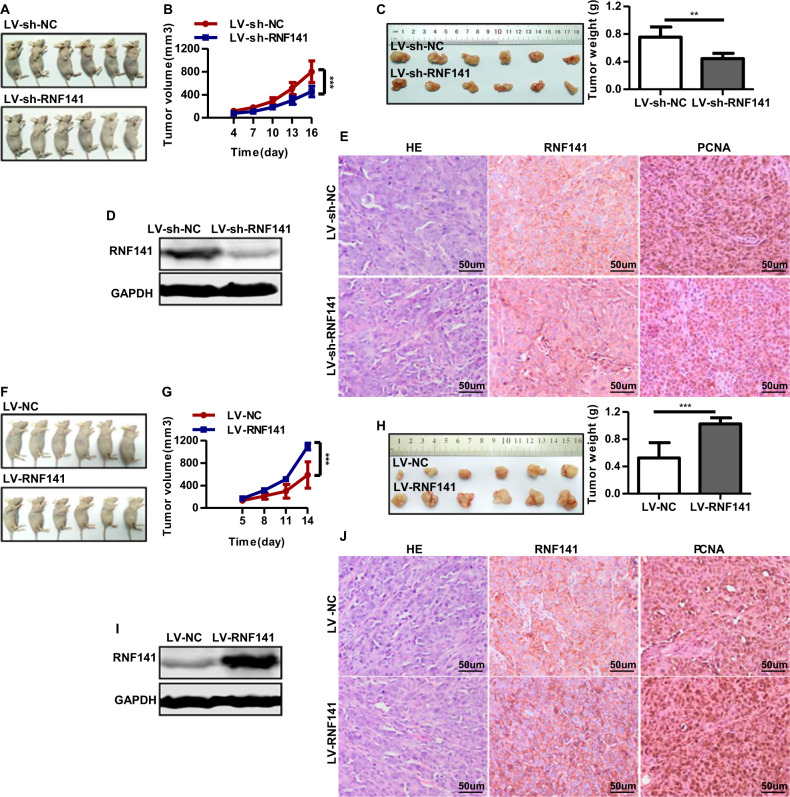
RNF141 involves in tumorigenesis in vivo. **A**–**E** Knockdown of RNF141 suppressed tumor growth in vivo. Images of LV-sh-NC or LV-sh-RNF141-transfected cell xenograft tumor models (**A**); Tumor growth curve (**B**) and average weight (**C**) were recorded and counted. The RNF141 protein level in xenograft tumors from LV-sh-NC or LV-sh-RNF141 group was confirmed by Western blot (**D**). The IHC staining (**E**) was applied to determine the expression of RNF141 and PCNA in LV-sh-NC or LV-sh-RNF141-transfected cell xenograft tumor. Scale bar, 50 μm. **F**–**J** RNF141 overexpression facilitated tumor growth in vivo. Xenograft tumor models of HCT116 cells transfected with LV-NC or LV-RNF141 were displayed (**F**), Tumor growth curve (**G**) and average weight (**H**) were recorded and counted. Western blot was performed to confirm RNF141 protein level in xenograft tumors from LV-NC or LV-RNF141 group (**I**). The IHC staining (**J**) was applied to determine the expression of RNF141 and PCNA in LV-NC or LV-RNF141-transfected cell xenograft tumor. Scale bar: 50 μm. Data represented mean ± SD. *n* = 6 each group. **P < 0.01, ***P < 0.001.

The above studies demonstrated that RNF141 promoted the tumorigenic potential of CRC cell in vitro. We next established a subcutaneous xenograft model to explore tumor-forming capacity of RNF141 in vivo. Nude mice injected with LV-sh-RNF141-infected HCT116 cells were found to have smaller volume and lower weight tumor than those injected with LV-sh-NC-infected cells at the assigned day (Fig. 5A, B, C). Meanwhile, overexpression of RNF141 showed the opposite effect that the subcutaneous tumors derived from LV-RNF141-infected HCT116 cells were larger in volume and weight than those derived from LV-NC-infected HCT116 cells (Fig. 5F, G, H). The RNF141 expression in xenograft tumors was confirmed by Western blot and immunohistochemistry staining. The results showed that RNF141 was rarely expressed in tumors derived from LV-sh-RNF141-infected HCT116 cells and highly expressed in tumors derived from LV-RNF141-infected HCT116 cells (Fig. 5D, I and E, the middle panel; Fig. 5J, the middle panel). In addition, PCNA immunohistochemistry staining was employed to evaluate the proliferative index of the above xenograft tumors. We found that RNF141 knockdown tumors had less percentage of PCNA-positive cells than control tumors (Fig. 5E, the right panel), indicating downregulation of RNF141 suppressed the cell proliferative ability. Conversely, the percentage of PCNA-positive cells in RNF141 overexpression tumors was much higher than that in control tumors (Fig. 5J, the right panel), suggesting that upregulation of RNF141 enhanced the cell proliferative ability.

### RNF141 induces KRAS activation via interacting with KRAS to increase its enrichment on the plasma membrane

**Fig. 6:**
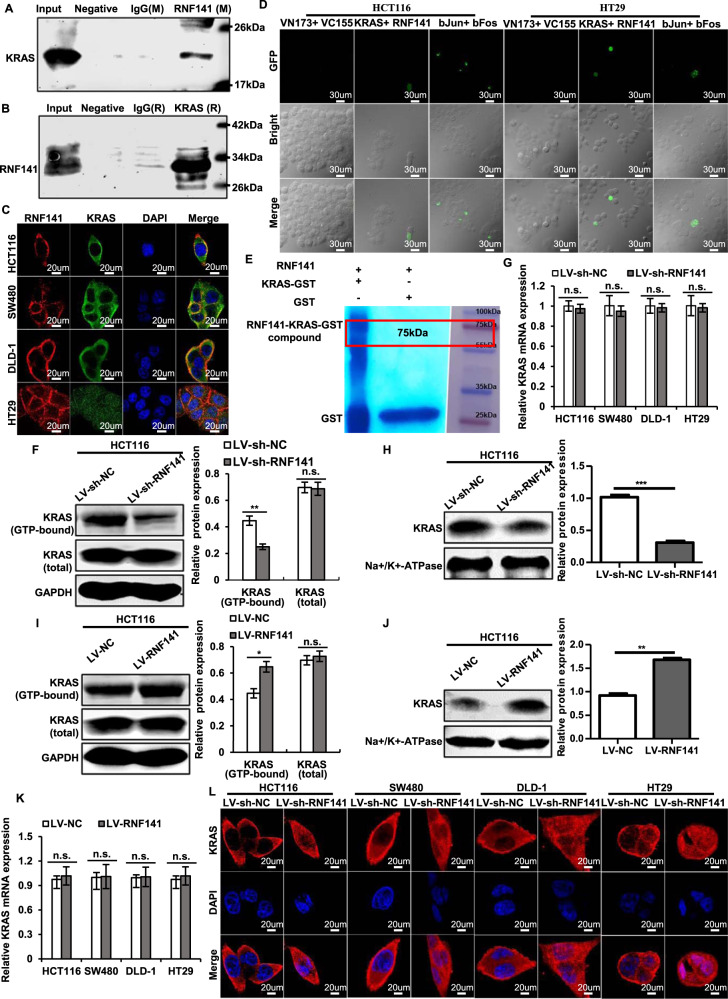
RNF141 interacts with KRAS and upregulates KRAS activity by promoting its membrane translocation. **A**, **B** Immunoprecipitation (IP) was conducted using RNF141 antibody (mouse derived) and followed by Western blot using KRAS antibody (rabbit derived) (**A**); meanwhile, IP using KRAS antibody and followed by Western blot using RNF141 antibody (**B**). IgG antibody (rabbit or mouse derived) was used as non-specific control. **C** The colocations between RNF141 and KRAS in CRC cells were determined by double immunofluorescence staining. Scale bar, 20 μm. **D** The direct visualization of RNF141 and KRAS interaction in HCT116 and HT29 cells were demonstrated by BiFC assay. bJunVN173 and bFosVC155 were used as positive control, VN173 and VC155 were used as negative control. **E** Detection of full-length RNF141 bound to KRAS-GST or GST in a GST pull-down assay. **F**, **I** Active KRAS was assessed by K-Ras Activation Assay Kit in HCT116 cell transfected with indicated lentivirus. Meanwhile, whole cell lysates were subjected to Western blot for total KRAS detection. **G**, **K** RNF141 mRNA levels were validated in HCT116, SW480, DLD-1, and HT29 cells by real-time PCR. **H**, **J** KRAS protein levels of membrane extraction in CRC cells transfected with indicated lentivirus was assessed by Western blot. Na+/K+-ATPase was used as membrane internal control. **L** The effect of RNF141 knockdown on KRAS membrane/cytoplasmic localization was visualized by IF assay. Scale bar, 20 μm. Bar graphs were presented as the mean ± SD from three independent experiments. **P < 0.01, ***P < 0.001. *n.s*. represents no significance.

To explore the mechanism by which RNF141 regulated colorectal tumorigenesis, we conducted a series of experiments. First, immunofluorescence staining showed that RNF141 was mainly localized in the cytoplasm and cell membrane in HCT116, SW480, DLD-1, and HT29 cells (Fig. s4A), which is consistent with the distribution of RNF141 in colorectal cancer tissues. To further verify the location of RNF141 in CRC cells, cell components of the cytoplasm and nucleus were extracted separately and proceeded for RNF141 measurement by Western blot. As shown in Fig. s4B, RNF141 was mainly expressed in the cytoplasm but rarely observed in the nucleus. Next, a combination of IP and LC-MS/MS were carried out to identify the protein interactions of RNF141. A total of 143 proteins were identified as candidates in both LV-NC and LV-RNF141 transfected HCT116 cells, and the genes encoding these proteins were annotated which are listed in Supplementary data 2. Here, we focused on KRAS owing to its relative high abundance in the interactome and definite oncogenic properties in CRC tumorigenesis [25, 26]. Herein we utilized Co-IP assays to further demonstrate the interaction between KRAS and RNF141. As a result, KRAS was immunoprecipitated by mouse RNF141, whereas no target protein was immunoprecipitated with the control mouse IgG and negative controls (Fig. 6A). Subsequently, as shown in Fig. 6B, RNF141 was immunoprecipitated by rabbit KRAS. Similarly, no such protein was immunoprecipitated with the control rabbit IgG and negative controls, confirming KRAS could interact with RNF141. In an attempt to purse whether KRAS bound to RNF141, co-localization experiments were carried out. As a result, Co-IF assay showed that KRAS co-localized with RNF141 and predominantly accumulated on the plasma membrane (Fig. 6C, yellow signal), To further determinate the endogenous interaction between RNF141 and KRAS, BiFC assay was carried out in CRC cells, and the results provided further support that there was indeed direct interaction between RNF141 and KRAS in vivo (Fig. 6D, Fig. s5A). Next, to identify the interaction domain of RNF141 binding to KRAS, we performed GST pull-down assay in an *E.coli* system. According to the RNF domain, RNF141 was divided into two peptides of 1H-RNF141 (1-144aa) and 2H-RNF141 (RNF domain, 145-230aa). Because the 1H-RNF141 peptide formed inclusion body, only 2H-RNF141 and RNF141 proteins were made the purification. The purified proteins were identified by SDS-PAGE and Western blotting analysis (Fig. s5B). As shown in Fig. 6E, RNF141 directly bound to KRAS-GST, but not GST, yet the 2H-RNF141 failed to interact with KRAS-GST (Fig. s5C). This experiment showed that the whole RNF141 peptide was indispensable to the direct interaction between RNF141 and KRAS.

It’s well known that the GTP-bounded KRAS is the active state and essential for cell differentiation, proliferation, and survival [27–29]. Then we examined the activated KRAS by KRAS Activation Assay Kit. As expected, the knockdown of RNF141 decreased the activated KRAS expression (Fig. 6F); in contrast, the overexpression of RNF141 increased the activated KRAS expression (Fig. 6I). However, neither RNF141 knockdown nor RNF141 overexpression had effect on total KRAS protein expression in the whole lysates (Fig. 6F, I). To clarify the effects that RNF141 exerted on KRAS expression, we tested KRAS mRNA by RT-PCR. Nevertheless, neither LV-sh-RNF141 nor LV-RNF141 group showed significant difference of KRAS mRNA expression compared with corresponding controls (Fig. 6G, K). It is reported that the KRAS activity is inextricably linked to its enrichment at the plasma membrane [30]. Next, membrane proteins were extracted and detected for the expression level of RNF141and KRAS by Western blot analysis. The results were in line with our speculation that the expression level of KRAS on the plasma membrane along with RNF141 knockdown in LV-sh-RNF141 group exhibited a significant reduction compared with control cells (Fig. 6H), and along with RNF141 overexpression in LV-RNF141 group showed a clear increase relative to control cells (Fig. 6J), which were further verified by IF assay (Fig. 6L, Fig. s5E). Additionally, we detected KRAS mutation of 64 CRC patients to analyze the expression of RNF141 in KRAS-mutant or KRAS-wildtype CRC tissues. We found that there were 24 KRAS-mutant CRC patients accounting for 37.5% in 64 cases (Supplementary Table 1) and no significant difference of RNF141 protein level between KRAS-mutant and KRAS-wildtype CRC tissues (*P* > 0.05) (Fig. s5D). Taken together, these results supported that RNF141 upregulated KRAS activity by elevating its enrichment at the plasma membrane, but not affecting KRAS expression.

### RNF141 regulates cell proliferation and apoptosis by KRAS

**Fig. 7:**
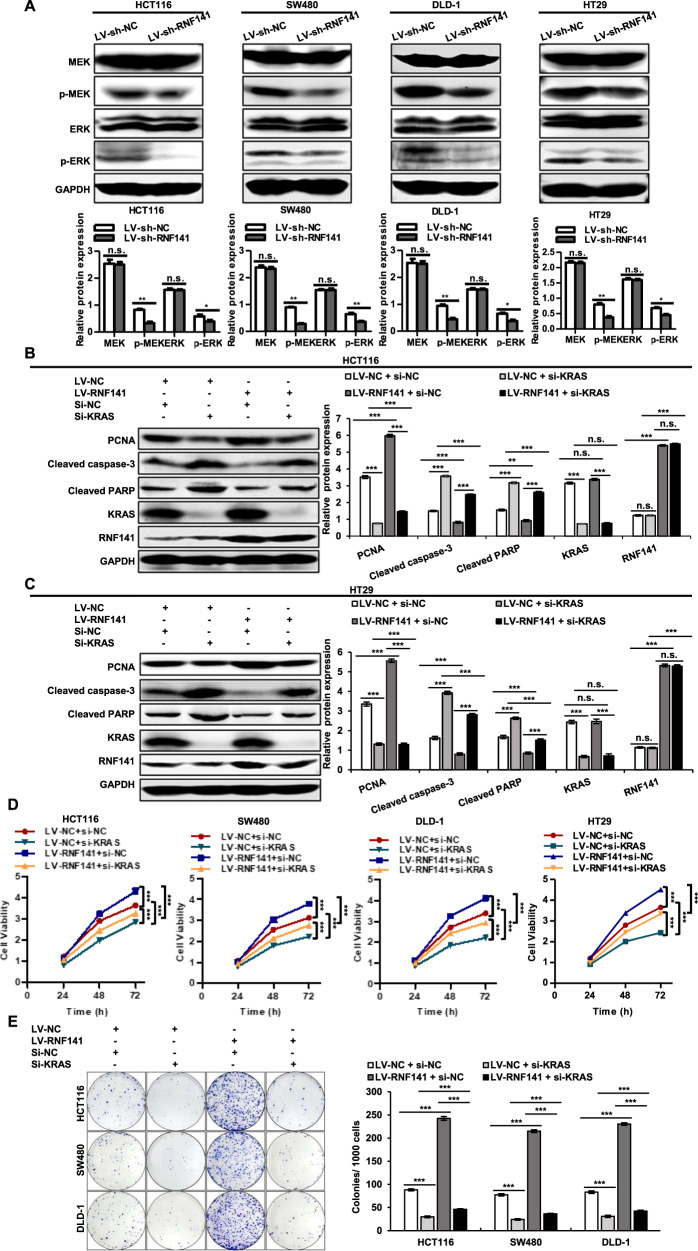
RNF141 regulates cell proliferation and apoptosis by KRAS. **A** The effects of RNF141 knockdown on protein levels of MEK, p-MEK, ERK, p-ERK was assessed by Western blot. **B**, **C** Protein levels of PCNA, Cleaved caspase-3, Cleaved PARP, KRAS, and RNF141 in HCT116 (**B**) and HT29 (**C**) cells co-transfected with indicated siRNA and lentivirus were validated by Western blot. **D**, **E** Cell proliferation of HCT116, SW480, DLD-1 cells co-transfected with indicated siRNA and lentivirus was examined by CCK-8 assay (**D**) and plate colony formation assay (**E**). Data were mean ± SD from three independent experiments, each performed in triplicate. **P < 0.01, ***P < 0.001.

Having established the promotive effect of RNF141 on KRAS activity, we next analyzed the role of RNF141 on KRAS signal pathway, MEK-ERK signaling. As shown in Fig. 7A, the knockdown of RNF141 decreased the p-MEK and p-ERK expression; in contrast, the overexpression of RNF141 increased the p-MEK and p-ERK expression (Fig. s6A). Neither knockdown nor overexpression altered MEK and ERK expression. We continued to silence KRAS in both LV-NC, and LV-RNF141 transfected CRC cells and carried out rescue experiment for KRAS. Western blot analysis showed that silence of KRAS inhibited RNF141-dependent proliferation and aggravated cell apoptosis determined by PCNA, cleaved caspase-3, and cleaved PARP (Figs. 7B, C, and s6B, C). Both CCK-8 assay and plate colony formation assay simultaneously exhibited that KRAS silence inhibited cell proliferation and partially attenuated promotive effect of RNF141 on proliferation (Fig. 7D, E). Accordingly, these data conformed that RNF141 modulated cell proliferation and apoptosis by KRAS.

### RNF141 interacts with LYPLA1 to promote KRAS membrane trafficking

**Fig. 8:**
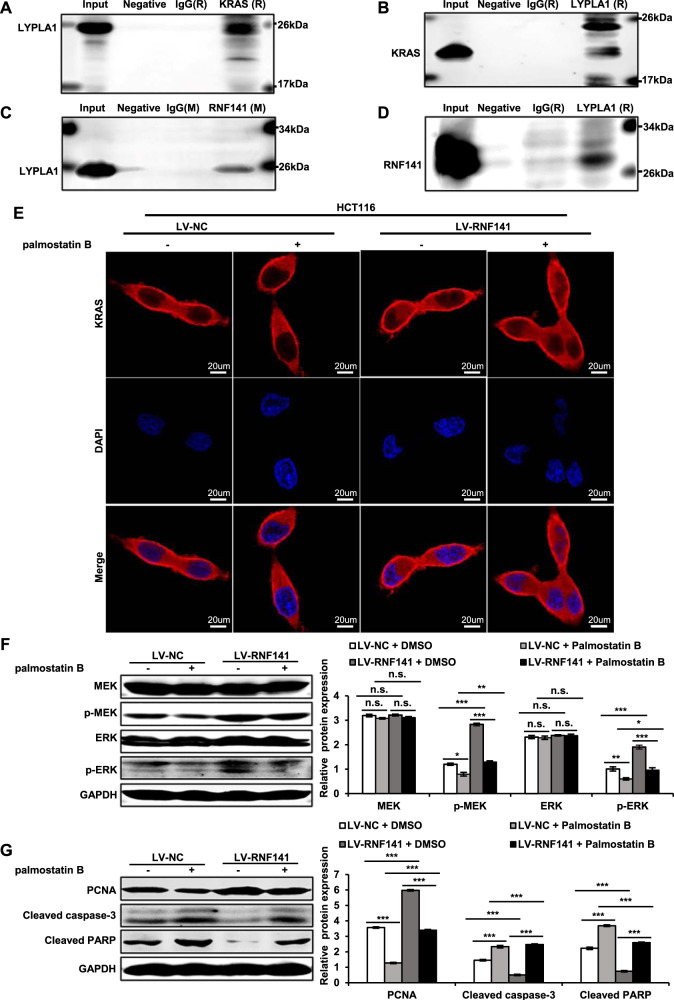
RNF141 interacted with LYPLA1 to promote KRAS membrane traffic. **A** Immunoprecipitation (IP) was conducted using KRAS antibody (rabbit derived) and followed by Western blot using LYPLA1 antibody (rabbit derived). **B** IP using LYPLA1 antibody and followed by Western blot using KRAS antibody. **C** IP using RNF141 antibody and followed by Western blot using LYPLA1 antibody. **D** IP using LYPLA1 antibody and followed by Western blot using RNF141 antibody. IgG antibody (rabbit or mouse derived) was used as non-specific control. **E** IF assay displayed the level of KRAS on the membrane after treatment with palmostatin B (50 μΜ, incubation 6 h) in HCT116 cells transfected with LV-NC and LV-RNF141. Scale bar, 20 μm. **F**, **G** Western blot analyses for KRAS downstream effectors (**F**) and PCNA and apoptotic markers (**G**) after treatment with palmostatin B (50 μΜ, incubation 6 h) in HCT116 cells transfected with LV-NC and LV-RNF141. Bar graphs were presented as the mean ± SD from three independent experiments. **P < 0.01, ***P < 0.001. *n.s*. represents no significance.

To explore how RNF141 facilitated KRAS membrane enrichment, we attempted to analyze the interaction between RNF141 and traffic proteins related to KRAS. Interestingly, we also found the traffic protein LYPLA1 (Lysophospholipase1, LYPLA1) also known as APT1 (acyl-protein thioesterase 1, APT1) in proteins database from LC–MS/MS (Supplementary data 2). Then co-IP assays were used to demonstrate the interactions among RNF141, KRAS, and LYPLA1. As shown in Fig. 8A–D, there were interactions among RNF141, KRAS, and LYPLA. RAS localization is not unchanged and RAS traffics between membrane and cytoplasm depends on its activation [31] and palmitoylation status [32]. As reported, LYPLA1 regulates Ras localization and signaling, and its inhibitor palmostatin B interferes the cellular acylation cycle at the level of depalmitoylation [32]. Then LV-NC or LV-RNF141 transfected CRC cells were treated with palmostatin B. IF assay showed that the treatment of palmostatin B caused a loss of KRAS membrane localization in HCT116 cell transfected with LV-NC or LV-RNF141, compared with DMSO treatment (Fig. 8E). Furthermore, Western blot analysis revealed that the treatment of palmostatin B not only inhibited the RNF141-dependent MEK-ERK signaling determined by p-MEK and p-ERK (Fig. 8F), but suppressed proliferation and aggravated cell apoptosis determined by PCNA, cleaved caspase-3, and cleaved PARP (Fig. 8G). The above mentioned results suggested that RNF141 promoted KRAS membrane traffic through interacting with LYPLA1.

## Discussion

Accumulative evidences reveal that many RNF finger proteins play either oncogenic or tumor-suppressor roles in colorectal tumorigenesis involving either ubiquitylation-dependent or ubiquitylation-independent effects [18, 33]. Due to RING finger domain, RNF141 is categorized as RING E3 ubiquitin ligase and its roles in cancer, especially colorectal cancer, remain largely unknown. To the best of our knowledge, this is the first study to comprehensively determine the biological function and mechanism of RNF141 in colorectal cancer. The results of the current study indicated that RNF141 was extensively upregulated in CRC tissues and the overexpression of RNF141 was positively correlated with high T stage. IHC study suggested that RNF141 was mainly expressed in the membrane and cytoplasm, and confirmed the location in CRC cells by IF assay, which is different from the early report that RNF141 was distributed in the nucleus in the testis [23]. It appeared that a different location of RNF141 in CRC cells might promote tumor progression through different patterns. Next, we undertook a series of in vitro and in vivo experiments to systematically investigate the effects of RNF141 on CRC progression. Our data showed that knockdown of RNF141 inhibited CRC cell proliferation, colony formation, migration, invasion, HUVEC tube formation and tumorigenicity, and induced cell apoptosis and cell cycle arrest, whereas the opposite results were witnessed in RNF141 overexpression assays. Therefore, our study first indicated that RNF141 functioned as an oncogene in colorectal tumorigenesis and might be a novel therapeutic target for CRC.

Subsequently, to further elucidate the underlying mechanism by which RNF141 exerted its functions in CRC, we combined IP and LC–MS/MS to screen out the target proteins that interacted with RNF141, and identified more than 30 cancer-related proteins in total 143 proteins that might have interaction with RNF141, including tumor-suppressor factors, such as KLF4, and oncogenic factors, such as KRAS, YY1, LYPLA1, and so on.

KRAS, a monomeric membrane-localized GTPases, is the most frequently mutated RAS isoform in various cancers, such as pancreatic adenocarcinoma (90%), colorectal cancer (45%) and lung adenocarcinoma (35%), and has been found to play pivotal role in oncogenesis [34, 35]. In the absence of activating mutations, KRAS may still contribute to tumorigenesis through gene amplification, overexpression, or upstream activation [36]. As extensively reported, KRAS triggered the CRC initiation and promoted cell proliferation, stimulated angiogenesis, and blocked cell apoptosis [37–39]. KRAS, because of its relative high abundance in the interactome and definite oncogenic properties, was selected as the target that RNF141 interacted with. Then, Co-IP assay, Co-IF and BiFC assay were performed and we found that there was a clearly defined interaction and co-location between RNF141 and KRAS in CRC cells, and GST pull-down assay showed full-length RNF141 could directly bind to KRAS. In the light of its characteristics that KRAS function as a “binary functional molecular switch” cycling between the inactive GDP-bound state and the active GTP-bound state [40–42], we determined active GTP-binding KRAS in CRC cells using a K-Ras Activation Assay Kit, and found that the overexpression of RNF141 raised the level of active GTP-bound KRAS and the knockdown of RNF141 in turn decreased the active GTP-bound KRAS. Meanwhile, Western blot analysis was utilized to examine total KRAS in whole cell lysates, yet neither enhanced RNF141 cells nor RNF141 knockdown cells showed difference of total KRAS in whole cell lysates when compared with their corresponding control cells. To confirm the above result, RT-PCR was further employed to detect the KRAS mRNA level and showed similar results. It was additionally verified by evidences that the overexpression of RNF141 increased the KRAS expression level of membrane extraction, whereas the knockdown of RNF141 exhibited the opposite effects, indicating that RNF141 increased the accumulation of KRAS on cell membrane via a mechanism independent of total KRAS alterations. Previous studies have revealed that membrane localization was inextricably required for KRAS transforming activity [43, 44], which might make a logical explanation for the above results. KRAS 4A, HRAS, and NRAS localizes to the plasma membrane through utilizing palmitoylation [34, 45, 46]. Interestingly, in our study, we also found the interaction among RNF141, KRAS, and LYPLA1. As reported, LYPLA1 manifests depalmitoylation to regulate RAS membrane association and its inhibitor palmostatin B moderates RAS membrane enrichment [32]. Our subsequent findings showed that the treatment of palmostatin B decreased KRAS membrane localization and perturbed the RNF141-dependent effects on KRAS downstream effectors in comparison to DMSO treatment. Taken together, it was sufficiently reasonable to suggest that the interaction between RNF141 and KRAS promoted KRAS activity via increasing its enrichment at plasma membrane, which was facilitated by the interaction between RNF141 and LYLPA1. Nevertheless, the exact mechanisms of whether RNF141 facilitates KRAS membrane enrichment in a ubiquitylation-dependent or ubiquitylation-independent manner still remains unclear. It was previously reported that mono-ubiquitin modification of KRAS at different specific site generated different impacts on protein–protein interactions and KRAS activity in a nucleotide-independent or nucleotide-dependent manner [47–50], which accounted for the intricacy of KRAS and reiterative failure of anti-KRAS therapies despite more than 30 years of research [12].

In conclusion, our study uncovered that the expression of RNF141 in CRC tissue was significantly upregulated, and closely associated with high T stage of CRC patient. Our systematic analysis revealed the in vivo and in vitro positive impacts of RNF141 on CRC. Mechanistically, RNF141 interacted with KRAS and promoted KRAS activity via increasing its enrichment on plasma membrane. Owing to the target proteins screened out by LC–MS/MS including more than 30 cancer-related proteins, further works are required to elaborate the interaction network of RNF141 that involves in the CRC tumorigenesis.

## Materials and methods

### Patients and samples

Fresh surgically resected tissue samples, including CRC tissues and matched adjacent normal tissues from 64 CRC patients who underwent complete surgical excision between January 2014 and December 2016, were randomly obtained from the Second Hospital of Hebei Medical University, Shijiazhuang, China. The diagnosis of CRC was confirmed independently by two pathologists. After excision, 64 pairs of CRC tissues and matched adjacent normal tissues were randomly selected, and every pair was divided into three portions: one portion was directly snap-frozen in liquid nitrogen for Western blot; another portion was immediately immersed in RNAstore reagent from Tiangen Biotech Co., Ltd (Beijing, China) and stored in −80 °C freezer for quantitative real-time polymerase chain reaction (RT-PCR); another one portion was fixed in 4% paraformaldehyde (PFA) for further paraffin embedding. This study was approved by the Ethics Committee of the Second Hospital of Hebei Medical University, and written informed consent for collecting and preserving samples and details was obtained from each patient before the study (Supplementary data 4).

### Histology and Immunohistochemistry

Human colorectal tissue samples and the follow-up xenograft tumor samples were fixed in 4.0% PFA, embedded in paraffin, and cut into two 5 μm sections. Sections were used for hematoxylin and eosin (H&E) staining and immunohistochemistry (IHC) according to the standard procedures [51]. The IHC staining protocol was briefly described as follows: the slides were routinely deparaffinized, rehydrated, subjected to antigen retrieval, and incubated in 3% hydrogen peroxide to block endogenous peroxidase. Subsequently, the slides were blocked and incubated with primary antibodies against RNF141 (1:30, Santa Cruz, USA), PCNA (1:100, Proteintech, China) at 4 °C overnight, and with polymer-HRP-conjugated anti-rabbit or anti-mouse secondary antibody the next day. Then, the section was stained with 3,3′-diaminobenzidine (DAB), counterstained with hematoxylin, dehydrated, and cover-slipped.

### RNA extraction and real-time PCR

Total RNA was extracted from tissues using TRIzol reagent (Tiangen). Complementary DNA (cDNA) was synthesized from total RNA through reverse transcription using PrimeScript^TM^ RT reagent kit from Takara Biomedical Technology (Beijing) Co., Ltd (Beijing, China). The cDNA was used for RT-PCR using the above PrimeScript^TM^ RT reagent kit on GeneAmp7300 RT-PCR System (Applied Biosystems, MS). GAPDH was used as an internal control. The specific primer sequences were designed as following: RNF141: Forward primer 5′-GTTGGTTCGAGAGAGTGGCT-3′, Reverse primer 5′-TGGTACAGACCACCCG TACA-3′; KRAS: Forward primer 5′-CGGCGGCGGAGGCAGCA-3′; Reverse primer 5′-CCCGCCGCCGCCTTCAGT-3′; GAPDH: Forward primer 5′-CAGGGGGGAGCCAAAAGGGTCA-3′, Reverse primer 5′-TGGGTGGCAGTGATGGCATGGA-3′.

### Cell culture

The human CRC cell lines HCT116, SW480, DLD-1, and HT29 were obtained from Shanghai Institute of Biochemistry and Cell Biology, Chinese Academy of Sciences (Shanghai, China). The HCT116 and HT29 cells were cultured in McCoy’s 5A Medium (Sigma-Aldrich, St. Louis, MO, USA) and SW480 cells and DLD-1 cells were cultured in RPMI 1640 medium (Gibco BRL, Rockville, MD, USA), all of which are supplemented with 10% FBS (Gibco BRL), 100 U/mL penicillin, and 100 μg/mL streptomycin at 37 °C in a humidified incubator with 5% CO_2_. HCT116, SW480, DLD-1, and HT29 cells were authenticated by Viva Cell Biosciences Ltd (Shanghai, China), and the reports of Cell Line Identification were presented in Supplementary data 1.

## Supplementary information

Supplementary methods

Supplementary figure legends

Supplementary table 1

Fig. s1

Fig. s2

Fig. s3

Fig. s4

Fig. s5

Fig. s6

Supplementary data 1

supplementary data 2

Supplementary data 3

Supplementary data 4

## References

[1] Bray F, Ferlay J, Soerjomataram I, Siegel RL, Torre LA, Jemal A (2018). Global cancer statistics 2018: GLOBOCAN estimates of incidence and mortality worldwide for 36 cancers in 185 countries. CA Cancer J Clin.

[2] Dekker E, Tanis PJ, Vleugels JLA, Kasi PM, Wallace MB (2019). Colorectal cancer. Lancet.

[3] Cancer Genome Atlas N. (2012). Comprehensive molecular characterization of human colon and rectal cancer. Nature.

[4] Inamura K (2018). Colorectal cancers: an update on their molecular pathology. Cancers.

[5] Lee DW, Han SW, Cha Y, Bae JM, Kim HP, Lyu J (2017). Association between mutations of critical pathway genes and survival outcomes according to the tumor location in colorectal cancer. Cancer.

[6] Lieu CH, Golemis EA, Serebriiskii IG, Newberg J, Hemmerich A, Connelly C (2019). Comprehensive genomic landscapes in early and later onset colorectal cancer. Clin Cancer Res.

[7] Amado RG, Wolf M, Peeters M, Van Cutsem E, Siena S, Freeman DJ (2008). Wild-type KRAS is required for panitumumab efficacy in patients with metastatic colorectal cancer. J Clin Oncol.

[8] Koveitypour Z, Panahi F, Vakilian M, Peymani M, Seyed Forootan F, Nasr Esfahani MH (2019). Signaling pathways involved in colorectal cancer progression. Cell Biosci.

[9] Martinelli E, Ciardiello D, Martini G, Troiani T, Cardone C, Vitiello PP (2020). Implementing anti-epidermal growth factor receptor (EGFR) therapy in metastatic colorectal cancer: challenges and future perspectives. Ann Oncol.

[10] Normanno N, Tejpar S, Morgillo F, De Luca A, Van Cutsem E, Ciardiello F (2009). Implications for KRAS status and EGFR-targeted therapies in metastatic CRC. Nat Rev Clin Oncol.

[11] Hobbs GA, Der CJ, Rossman KL (2016). RAS isoforms and mutations in cancer at a glance. J Cell Sci.

[12] Papke B, Der CJ (2017). Drugging RAS: know the enemy. Science.

[13] Senft D, Qi J, Ronai ZA (2018). Ubiquitin ligases in oncogenic transformation and cancer therapy. Nat Rev Cancer.

[14] Venuto S, Merla G (2019). E3 ubiquitin ligase TRIM proteins, cell cycle and mitosis. Cells.

[15] Jaworska AM, Wlodarczyk NA, Mackiewicz A, Czerwinska P (2020). The role of TRIM family proteins in the regulation of cancer stem cell self-renewal. Stem Cells.

[16] Wang XW, Wei W, Wang WQ, Zhao XY, Guo H, Fang DC (2014). RING finger proteins are involved in the progression of barrett esophagus to esophageal adenocarcinoma: a preliminary study. Gut Liver.

[17] Zhu J, Zhao C, Zhuang T, Jonsson P, Sinha I, Williams C (2016). RING finger protein 31 promotes p53 degradation in breast cancer cells. Oncogene.

[18] Liu L, Wong CC, Gong B, Yu J (2018). Functional significance and therapeutic implication of ring-type E3 ligases in colorectal cancer. Oncogene.

[19] Veggiani G, Gerpe MCR, Sidhu SS, Zhang W (2019). Emerging drug development technologies targeting ubiquitination for cancer therapeutics. Pharm Ther.

[20] Jiang X, Charlat O, Zamponi R, Yang Y, Cong F (2015). Dishevelled promotes Wnt receptor degradation through recruitment of ZNRF3/RNF43 E3 ubiquitin ligases. Mol Cell.

[21] Koo BK, Spit M, Jordens I, Low TY, Stange DE, van de Wetering M (2012). Tumour suppressor RNF43 is a stem-cell E3 ligase that induces endocytosis of Wnt receptors. Nature.

[22] Geng R, Tan X, Wu J, Pan Z, Yi M, Shi W (2017). RNF183 promotes proliferation and metastasis of colorectal cancer cells via activation of NF-kappaB-IL-8 axis. Cell Death Dis.

[23] Song H, Su D, Lu P, Yang J, Zhang W, Yang Y (2008). Expression and localization of the spermatogenesis-related gene, Znf230, in mouse testis and spermatozoa during postnatal development. BMB Rep.

[24] Deng W, Sun H, Liu Y, Tao D, Zhang S, Ma Y (2009). Molecular cloning and expression analysis of a zebrafish novel zinc finger protein gene rnf141. Genet Mol Biol.

[25] Castagnola P, Giaretti W (2005). Mutant KRAS, chromosomal instability and prognosis in colorectal cancer. Biochim Biophys Acta.

[26] Smakman N, Borel Rinkes IH, Voest EE, Kranenburg O (2005). Control of colorectal metastasis formation by K-Ras. Biochim Biophys Acta.

[27] Geyer M, Wittinghofer A (1997). GEFs, GAPs, GDIs and effectors: taking a closer (3D) look at the regulation of Ras-related GTP-binding proteins. Curr Opin Struct Biol.

[28] Levinson AM, McGee JH, Roberts AG, Creech GS, Wang T, Peterson MT (2017). Total chemical synthesis and folding of All-l and All-d variants of oncogenic KRas(G12V). J Am Chem Soc.

[29] Wang Y, Kaiser CE, Frett B, Li HY (2013). Targeting mutant KRAS for anticancer therapeutics: a review of novel small molecule modulators. J Med Chem.

[30] Schmick M, Kraemer A, Bastiaens PI (2015). Ras moves to stay in place. Trends Cell Biol.

[31] Prior IA, Hancock JF (2001). Compartmentalization of Ras proteins. J Cell Sci.

[32] Dekker FJ, Rocks O, Vartak N, Menninger S, Hedberg C, Balamurugan R (2010). Small-molecule inhibition of APT1 affects Ras localization and signaling. Nat Chem Biol.

[33] Hoeller D, Dikic I (2009). Targeting the ubiquitin system in cancer therapy. Nature.

[34] Adjei AA (2001). Blocking oncogenic Ras signaling for cancer therapy. J Natl Cancer Inst.

[35] Downward J (2003). Targeting RAS signalling pathways in cancer therapy. Nat Rev Cancer.

[36] Friday BB, Adjei AA (2005). K-ras as a target for cancer therapy. Biochim Biophys Acta.

[37] Bos JL, Fearon ER, Hamilton SR, Verlaan-de Vries M, van Boom JH, van der Eb AJ (1987). Prevalence of ras gene mutations in human colorectal cancers. Nature.

[38] Haigis KM, Kendall KR, Wang Y, Cheung A, Haigis MC, Glickman JN (2008). Differential effects of oncogenic K-Ras and N-Ras on proliferation, differentiation and tumor progression in the colon. Nat Genet.

[39] Shirasawa S, Furuse M, Yokoyama N, Sasazuki T (1993). Altered growth of human colon cancer cell lines disrupted at activated Ki-ras. Science.

[40] Boguski MS, McCormick F (1993). Proteins regulating Ras and its relatives. Nature.

[41] Donovan S, Shannon KM, Bollag G (2002). GTPase activating proteins: critical regulators of intracellular signaling. Biochim Biophys Acta.

[42] Schubbert S, Shannon K, Bollag G (2007). Hyperactive Ras in developmental disorders and cancer. Nat Rev Cancer.

[43] Jackson JH, Li JW, Buss JE, Der CJ, Cochrane CG (1994). Polylysine domain of K-ras 4B protein is crucial for malignant transformation. Proc Natl Acad Sci USA.

[44] Kato K, Cox AD, Hisaka MM, Graham SM, Buss JE, Der CJ (1992). Isoprenoid addition to Ras protein is the critical modification for its membrane association and transforming activity. Proc Natl Acad Sci USA.

[45] McCormick F (2015). KRAS as a therapeutic target. Clin Cancer Res.

[46] Tsai FD, Lopes MS, Zhou M, Court H, Ponce O, Fiordalisi JJ (2015). K-Ras4A splice variant is widely expressed in cancer and uses a hybrid membrane-targeting motif. Proc Natl Acad Sci USA.

[47] Baietti MF, Simicek M, Abbasi Asbagh L, Radaelli E, Lievens S, Crowther J (2016). OTUB1 triggers lung cancer development by inhibiting RAS monoubiquitination. EMBO Mol Med.

[48] Dohlman HG, Campbell SL (2019). Regulation of large and small G proteins by ubiquitination. J Biol Chem.

[49] Jura N, Scotto-Lavino E, Sobczyk A, Bar-Sagi D (2006). Differential modification of Ras proteins by ubiquitination. Mol Cell.

[50] Sasaki AT, Carracedo A, Locasale JW, Anastasiou D, Takeuchi K, Kahoud ER (2011). Ubiquitination of K-Ras enhances activation and facilitates binding to select downstream effectors. Sci Signal.

[51] Tan Z, Gao L, Wang Y, Yin H, Xi Y, Wu X (2020). PRSS contributes to cetuximab resistance in colorectal cancer. Sci Adv.

