# Supplementary material for: RNF141 interacts with KRAS to promote colorectal cancer progression
Source: Oncogene. 2021 Aug 3;40(39):5829–42. doi: 10.1038/s41388-021-01877-4 (PMC8484013; doi:10.1038/s41388-021-01877-4)
Supplement: Supplementary file 1 — Supplementary methods [file 41388_2021_1877_MOESM1_ESM.docx]

**Materials and methods**

**Cell transfection and lentivirus infection.** U6-shRNA-Luciferase17-Puro (LV16), pGLV-EF1aF-H-RNF141-GFP-Puro (LV5) and their corresponding control lentiviral vectors were purchased from GenePharma Tech (Shanghai, China). RNF141 shRNA target sequences were as follows: 5'-GGCATCACGGATCATGAAT-3', and a non-specific scrambled sequence ：5'-TTCTCCGAACGUGUCACGT-3' was used as a negative control (NC). HCT116, SW480 and DLD-1 cell lines were transfected with RNF141 short hairpin RNA (shRNA) lentiviral (LV-sh-RNF141) vectors and the NC lentiviral (LV-sh-NC) vectors to downregulate RNF141 expression. HCT116, SW480, DLD-1 and HT29 cell lines were transfected with RNF141 cDNA lentiviral (LV-RNF141) vectors and the corresponding NC (LV-NC) vectors. The stable transfected cells with RNF141 overexpression or knockdown and their corresponding control cells depending on antibiotic-resistance were screened by adding puromycin to the culture medium for 7 days.

To silence KRAS expression, siRNA against human KRAS (GenePharma) was transfected into cells transfected with LV-RNF141 or LV-NC. The siRNA sequences are listed as follows: si-KRAS: sense 5'-GAUGAUGCCUU-CUAUACAUTT-3', antisense 5'-AUGUAUAGAAGGCAUCAUCTT-3'; Negative control (NC) sequence: sense 5'-UUCUCCGAACGUGUCACGUTT-3', antisense 5'-ACGUGACACGUUCGGAGA-ATT-3'. The transduction was conducted according to the specific manufacturer’s instructions. The efficiency of cell transfection and infection was confirmed by western blot.

**Western blot.** Total tissue or cell proteins were extracted using high-performance RIPA lysis buffer (Solarbio Science &Technology Co., Ltd, Beijing, China); nuclear and cytoplasmic proteins were extracted using Nuclear/Cytosol Fractionation Kit (Solarbio). Membrane proteins were extracted using membrane protein extraction kit (BestBio Science, Shanghai, China). The concentrations of protein were quantified using Pierce BCA Protein Assay Kit (Thermo Scientific, Rockford, IL), and protein denaturation was through boiling for 8 min in SDS loading buffer. Equal amounts of denatured proteins from each sample were added to sodium dodecyl sulphate–polyacrylamide gel (SDS–PAGE) and transferred onto polyvinylidene difluoride (PVDF) membranes. The membranes were blocked with 5% [defatted](javascript:;) milk on the shaker for 1 hour at room temperature and subsequently incubated overnight at 4°C with the primary antibodies. The membranes were washed in TBST for 30 minutes the next day and incubated with fluorescence-conjugated secondary antibodies (LI-COR Biosciences, Lincoln, NE, USA) for 1 h at room temperature. The bands were developed by an Odyssey infrared imaging system (LI-COR Biosciences), and quantified using densitometry by IPwin32 software (Media Cybernetics, Bethesda, MD, USA), and GAPDH served as an internal control. The primary antibodies chosen in this assay included rabbit anti-RNF141(1:500, Proteintech, Wuhan, China), mouse anti-RNF141 (1:200, Santa Cruz, CA, USA ), anti-PCNA (1:2000, Proteintech ), anti-cleaved caspase-3 (1:100, Cell Signaling Technology, Danvers, MA, USA), anti-cleaved PARP (1:500, Proteintech), anti-KRAS (1:1000, Proteintech), anti-p-MEK(1:1000, Abways, Shanghai, China), anti-ERK(1:1000, Abways, Shanghai, China), anti-p-ERK(1:500, Abways, Shanghai, China), anti-Na+/K+-ATPase(1:5000, Abways, Shanghai, China), anti-LYPLA1 (1:2000, Abways, Shanghai, China) and anti-GAPDH(1:5000, Abways, Shanghai, China) .

**CCK-8 viability assay.** Cell viability was assessed by the Cell Counting Kit-8 (CCK-8) (Dojindo Laboratories, Kumamoto, Japan). The procedure was as follows: in brief, cells at a density of 5.0 × 10^4^ cells/mL maintained in serum-free medium were seeded into 96-well plates with a final volume of 100 μL per well. After being cultured for 24, 48 and 72 h, the culture medium in indicated wells of 96-well plates was removed and substituted by 100 μL of CCK-8 diluent that CCK-8 reagent was diluted with serum-free medium at the ratio of 1:10. Then the plates were incubated for 2.5 h at 37°C in the incubator and the absorbance at 450 nm was measured by a microplate reader (BioTek, Winooski, VT, USA).

**Colony formation assay.** The plate colony formation assay was conducted as follows: HCT116, SW480,DLD-1 and HT29 cells with RNF141 stable overexpression or knockdown through lentivirus transfection were seeded (1000 cells per well, 2000 cells per well, 1000 cells per well and 1000cells per well, respectively) in a 6-well plate and then cultured for 10 days during which the culture medium was refreshed every 3 days. The plates containing colonies were then washed with PBS, fixed with 4% paraformaldehyde for 15 minutes, and stained with Giemsa solution for 10 minutes. Distinct colonies with at least 50 cells were manually counted and averaged over triplicate wells. The soft agar assays were performed as follows: the wells of 6-well plates were coated with 1.5 mL of the mixture containing equal volumes of 1.2% sterile agar and 2X medium supplemented with 20% FBS and 2% antibiotics. Subsequently, cells mentioned above were resuspended in 1.5 ml of the mixture containing an equal volume of 0.6% sterile agar and 2X medium supplemented with 20% FBS and 2% antibiotics and then seeded on top of 0.6% agar in the coated well. Individual colonies containing more than 10 cells were calculated.

**Cell cycle assay.** The indicated CRC cells mentioned above were seeded into 6-well plates and harvested after incubation with serum-free medium for 48h. Next, the cells were washed twice with one media volume of ice-cold phosphate buffered saline (PBS), and then fixed in precooled 70% ethanol at 4°C for 24 hours. Each cell sample was stained with about 0.5mL PI/RNase Staining Solution (Beyotime Biotechnology, Shanghai, China) at 37°C for 30 minutes. The cell cycle distribution was analyzed by a FACSVerse flow cytometer in combination with ModFit LT 5.0 software (BD Biosciences, San Diego, CA, USA).

**Xenograft tumor growth models.** Five-week-old male BALB/c nude mice were purchased from Beijing Vital River Laboratory Animal Technology Co., Ltd (Beijing, China) and were raised in the specific pathogen-free animal laboratory of the Fourth Hospital of Hebei Medical University, Shijiazhuang, China. A suspension of HCT116 cells transfected with LV-sh-RNF141 or LV-sh-NC (2 × 10^7^ cells in 150μL PBS per mouse) were injected subcutaneously into the dorsal right flank of nude mice. To comprehensively investigate the role of RNF141 on tumorigenicity *in vivo*, HCT116 cells transfected with LV-RNF141 or LV-NC (1.5 × 10^7^ cells in 150μL PBS per mouse) were also injected to generated subcutaneous xenograft tumors. The mice were randomly divided into experimental or control group and each group included six mice . Tumors were measured every three days using a vernier caliper, and tumor volume was calculated according to a modified ellipsoidal formula:$\text{ }\frac{\text{1}}{\text{2}}$length × width^2^ . The mouse models of which xenograft tumors derived from HCT116 cells transfected with LV-sh-RNF141 or LV-sh-NC were euthanized on the 16th day, and of which xenograft tumors derived from HCT116 cells transfected with LV-RNF141 or LV-NC were euthanized on the 14th day. Then tumors were completely resected and weighed. Subsequently, tumor tissues were reserved for western blot or formalin-fixed and then paraffin-embedded for H&E and IHC analysis. The experiments were conducted according to the relevant local and institutional guidelines in compliance with the Chinese Animal Protection Act (supplementary data 3).

**Cell migration and invasion assay.** For wound-healing assays, after the cell density reached 80-90% confluence, cell monolayers were straightly scratched by a sterile 200 μL pipette tip to generate a linear wound and then incubated in serum-free medium. Next cells were imaged by the phase-contrast microscope after scratching for 0 and 48 h, respectively. The average distance migrated by cells was measured between the two boundaries of a cellular wound. Each experiment was conducted at least in triplicate.

Transwell assays were conducted to assess cell migration and invasion. Matrigel (BD Biosciences, San Diego, CA, USA) was unfrozen at 4°C overnight and the 24-well transwell plates (8 μm pore size, Corning Costar, Cambridge, MA, USA) were precooled at -20°C for 20 minutes in advance. Fifty microliter Matrigel was diluted in 400 μL serum-free medium (dilution ratio: 1:8), and then the diluted Matrigel ( 60μL) was used to coat the polycarbonate membrane of 24-well transwell inserts in the invasion assay. After starvation of 12 hours, a total of 1×10^6^ cells per well were resuspended in 0.5mL serum-free medium and seeded into the upper chamber. Point six milliliter McCoy’s 5A or RPMI-1640 medium with 20% FBS as a chemoattractant was added to the lower chamber. After 24 hours, cells that invaded through the Matrigel membrane were fixed in 4.0% paraformaldehyde, stained with 0.1% crystal violet, and then counted under an inverted microscope. For the migration assay, 5×10^5^ cells per well were loaded into the upper chambers without Matrigel coating, and then incubated for 12 hours. Subsequently, the assay was conducted as for the invasive assay.

**Tube formation assay.** HUVECs (5×10^5^cells per well) were plated in 24-well plates pre-coated with Matrigel (BD Biosciences) (200 μL per well ,diluted to 1:1 in serum-free medium ) and incubated for 12 h in indicated conditioned medium[^9^](#_ENREF_9). The tube formation was observed with a phase contrast microscope (Olympus, Tokyo, Japan) at × 40 magnification.

**Apoptosis assays.** After starvation for 48 hours in a serum-free medium, cells were harvested and washed with ice-cold PBS twice. Flow cytometry was conducted using eBioscience^TM^ Annexin V Apoptosis Detection Kit (eBioscience, San Diego, CA, USA) to determine apoptotic cells according to the manufacturer’s instructions. Annexin V stainning cells were recognized as apoptotic cells. Data were analyzed by FlowJo-V10 software (FlowJo, Version X; TreeStar, Ashland, OR, USA).

Furthermore, the terminal deoxynucleotidyl transferase-mediated deoxyuridine triphosphate nick-end labeling (TUNEL) assay was employed to analyze cell apoptosis. The staining protocol was performed following the manufacturer’s guidelines provided by One-step TUNEL apoptosis assay kit (Beyotime, Shanghai, China). The percentage of TUNEL-positive cells that exhibited distinct purple nuclear staining out of the total number of cells was determined as the apoptosis index.

**Immunofluorescence assays.** Immunofluorescence was performed to determine the location and interaction of target proteins. The experiment procedure was briefly described as follows: cells grown on the glass coverslips were fixed with 4% PFA for 15 minutes, washed with PBS three times for 5 minutes each time on the shaker, permeabilized with PBS containing 0.3% Triton X-100 for 10 minutes and then blocked with 10% normal goat serum for 20 minutes. Next, the cells were incubated with mouse anti-RNF141(1:50, Santa Cruz, Dallas, TX, USA) and rabbit anti-KRAS (1:200, Proteintech, Rosemont, IL, USA) at 4 °C overnight. After the coverslips were rinsed in PBS three times the next day, cells on coverslips were blotted with secondary fluorescent antibody (FITC goat anti-rabbit IgG, 1:100, and CY3 goat anti-mouse IgG, 1:100, Beyotime) for 1 hour at room temperature. Following rinses in PBS for three times, cells were counterstained with 4, 6-diamidino-2-phenylindole (DAPI, Beyotime) and then photographed by a laser scanning confocal microscope (Olympus, Tokyo, Japan).

**IP and LC-MS/MS.** For endogenous immunoprecipitation (IP), fresh cells pellets were lysed in cold lysis buffer (20 mM Tris-HCl pH 7.5, 100 mM NaCl, 20Mm KCl, 1.5 mM MgCL2, 0.5% NP-40, 0.5M cocktails of proteinase and phosphatase inhibitors), incubated on ice for 30 minutes during which cell lysates were vortexed for 15 seconds every 5 minutes. Then cell lysates were centrifuged at 10,000 × g for 10 min to remove cell debris and incubated with protein A/G agarose beads (Beyotime) to preclear nonspecific binding. Subsequently, the precleared lysates were incubated with the primary antibodies, following with gentle agitation overnight at 4 °C and incubation with Protein A/G magnetic beads for 2 hours at 4 °C the next day. The primary antibodies include mouse anti-RNF141 (1:20, Santa Cruz), rabbit anti-RNF141 (1:50, Proteintech), rabbit anti-KRAS (1:50, Proteintech), rabbit anti-LYPLA1 (1:100, Abways) and normal rabbit or mouse IgG (homologous control antibody, 1:500, Beyotime). After incubation, the beads we recollected with a magnetic stand and washed five times with IP washing buffer (20 mM Tris-HCl pH 7.5, 200 mM NaCl, 20 mM KCl, 1.5 mM MgCL2, 0.5% NP-40). The IP beads were resuspended in 2X SDS loading buffer (Beyotime) and boiled for 8 minutes to release the bound proteins, which were subsequently resolved by SDS-PAGE, followed by a western blot analysis as described above.

Liquid chromatography-tandem mass spectrometry (LC-MS/MS) was performed to detect the potential interacting proteins. The protocol was described as follows: briefly, the IP beads mentioned above was washed twice with ice-cold PBS at 4 °C and accompanied with each wash removed by centrifugation. Then each bead sample was resuspended in SDT lysis buffer (100 mM Tris-HCl, 100 mM DTT, 4% SDS) and boiled for 5 minutes. After centrifugation, the supernatant was obtained, of which the proteome was digested by filter aided sample preparation( FASP). The peptides after digestion were desalted and quantified by estimating the absorbance at 280 nm. Subsequently, the desalted peptides were used for LC-MS/MS analysis using Easy nLC^TM^ 1200 instrument (Thermo Fisher Science, Waltham, MA, USA). All LC-MS/MS data were identified using MaxQuant 1.6.1.0 software against Uniprot Protein database.

**Bimolecular Fluorescence Complementation (BiFC) assay**. BiFC assay is a method used to directly visualize protein-protein interaction in vivo using live-cell imaging[^28^](#_ENREF_28). The three pairs of plasmids (BiFC-KRAS-VN173 and BiFC-RNF141-VC155, positive control plasmids (BiFC-bJunVN173 and BiFC-bFosVC155), and negative control plasmids (BiFC-VN173 and BiFC-VC155) ) were purchased from LC-Bio (Hangzhou, China). Log-phase growing cells were seeded on the glass coverslips and then incubated overnight for proper cell attachment and expansion in a 37 °C cell culture incubator. After incubation, the cells were rinsed twice with antibiotic-free medium and then co-transfected the paired plasmids with Lipofectamine 2000. The transfection medium was replaced with complete medium 6h post-transfection, and the cells were then photographed in 12h after transfection by a laser scanning confocal microscope (Olympus, Tokyo, Japan).

Glutathione-S-transferase (GST) pull-down assay. The plasmids for KRAS-GST , RNF141, 1H-RNF141 (1-144aa) and 2H-RNF141 (RNF domain, 145-230aa) were transfected into E. coli. The preparation and purification of the fusion proteins were commissioned to LC Biotech Co., Ltd. (Hangzhou, China) . Approximately 200 µg of GST and KRAS-GST fusion protein was immobilized in 80 µL of glutathione agarose and equilibrated before being incubated together at 4 ℃ for 2h with gentle rocking motion. Approximately 300 µg of RNF141 and 2H-RNF141 protein was added to the immobilized GST and KRAS-GST after 3 washes with wash buffer. The two fusion proteins were incubated overnight at 4°C under gentle rotation. The bound proteins were eluted with elution buffer (10 mM glutathione in PBS, pH 8.0) and analyzed by native polyacrylamide gel electrophoresis.

Detection of KRAS mutation. DNA was extracted from frozen CRC tissues using a DNA Extraction kit (Tiangen) according to the manufacturer's instructions. All primer sequences are listed as follows: Exon 2 forward primer: CCAGACTGTGTTTGTCCCTTC, Exon 2 reverse primer: TTTAAACCCACCTATAATGGTG; Exon 3 forward primer : CCA- GACTGTGTTTCTCCCTT, Exon 3 reverse primer: CACAAAGAA- AGCCCTCCCA; Exon 4 forward primer : TGATTTGCAGAAAACAGAT; Exon 4 reverse primer: GACACAAAACAGGCTCAGGA. Primers were synthesized by Sangon Biotech Co., Ltd. (Shanghai, China). PCR reactions were performed to amplify DNA. The PCR products were commissioned to Sangon Biotech for purification and Sanger sequencing. and the sequencing results were analyzed.

**K-Ras Activation Assay.** The active GTP-bound form of Ras was analyzed using a K-Ras Activation Assay Kit (Cell Biolabs; San Diego, CA, USA) according to the manufacturer's instructions. After starvation for 48 hours in serum-free medium, cells were washed twice with ice-cold PBS, harvested by scraping with a cell scraper, and lysed in ice-cold 1X Assay/Lysis Buffer on ice for 20 minutes. After centrifugation (14,000 × g) for 10 minutes at 4°C, the supernatant was collected, and then protein concentrations of cell lysates were quantified as described above. Each sample containing equivalent amounts of protein (1.0 mg) was incubated with 40 μL Raf1-RBD-agarose beads at 4°C for 1 hour with gentle agitation to pull down activated GTP bound Ras. Then the beads were pelleted by transient centrifugation, washed three times with 1X Assay Buffer and then resuspended in 40 μL 2X reducing SDS-PAGE sample buffer. Subsequently, after denaturation by boiling and centrifugation, supernatant samples including KRAS immunoblot positive control were analyzed by western blot using the supplied KRAS-specific polyclonal antibody. Besides, whole-cell lysates (50 µg) were also applied in western blot analysis in the same manner for the detection of total K-Ras.

**Statistical analysis.** Statistical analysis was accomplished using IBM SPSS Statistics 23.0. Student’s t-test or two-way ANOVA was carried out to assess data between two groups, and the values are shown as the mean ± standard deviation (SD). All statistical tests were two-tailed, and *P* < 0.05 was defined as a statistically significant difference.
