# Supplementary material for: RNF141 interacts with KRAS to promote colorectal cancer progression
Source: Oncogene. 2021 Aug 3;40(39):5829–42. doi: 10.1038/s41388-021-01877-4 (PMC8484013; doi:10.1038/s41388-021-01877-4)
Supplement: Supplementary file 2 — Supplementary figure legends [file 41388_2021_1877_MOESM2_ESM.docx]

**Supplementary figure legends**

**Fig. s1 Overexpression of RNF141 promotes cell proliferation and facilitates G1/S transition in vitro.** (A and B) Efficiency of RNF141 overexpression (A) and its effects on protein expression of PCNA (B) in CRC cells were examined by western blot. (C-E) Cell proliferation were further monitored by CCK-8 assays (C), plate colony formation assay (D) and soft agar assay (E). Scale bar, 500 μm. (F) Excess RNF141 facilitated the G1/S transition. * *P* < 0.05, ** *P* < 0.01, *** *P* < 0.001 *vs* indicated group. *n.s.* indicates no significance.

**Fig. s2 RNF141 overexpression inhibits cell apoptosis.** (A and B) Cell apoptosis was assessed by Annexin V-APC/PI staining (A) and TUNEL technology, Scale bar, 100 μm (B). (C) Activated apoptosis-related proteins cleaved caspase-3 and PARP were evaluated by western blot. All values were the mean ± S.D values from three independent experiments, * *P* < 0.05, ** *P* < 0.01, *** *P* < 0.001.

**Fig. s3 Enhanced RNF141 accelerates cell migration and invasion and facilitates the HUVEC tube formation in vitro.** (A) The effect of RNF141 overexpression on cell migration was assessed by wound-healing assay. Scale bar, 500 μm. (B and C) The migratory and invasive capacity of HCT116, SW480, DLD-1 and HT29 transfected with indicated lentivirus was detected by transwell and invasion assay. Scale bar, 100 μm. (D) In vitro HUVEC tube formation assay was carried out. Indicated lentivirus-transfected cancer cells were cultured for 24 h in serum-free media, and then conditioned media were collected. HUVEC cells were incubated in conditioned media. After 12 h, tube formation was measured. Scale bar, 1mm. Data were the mean ± S.D. from three independent experiments, *** *P* < 0.001 *vs* indicated groups.

**Fig. s4 RNF141 mainly locates in cytoplasm and plasma membrane.** (A) Representative immunofluorescence staining of RNF141 in HCT116, SW480,DLD-1 and HT29 cells. Scale bar, 20 μm. (B) RNF141 protein of cytoplasm and cell nucleus in HCT116 cell transfected with lentivirus RNF141 or NC (negative control) was extracted and analyzed by western blot. Lamin B was used as nuclear internal control and GAPDH as cytoplasm internal control.

**Fig. s5 RNF141 interacts with KRAS and promotes its membrane translocation.** (A) The direct visualization of RNF141 and KRAS interaction in SW480 and DLD-1 cells were demonstrated by BiFC assay. bJunVN173 and bFosVC155 were used as positive control, VN173 and VC155 were used as negative control. (B) KRAS-GST, RNF141 and 2H-RNF141 proteins expressed in an *E. coli* system were detected by SDS-PAGE. (C) Detection of 2H-RNF141 bound to KRAS-GST and GST in a GST pull-down assay. (D) The expression of RNF141 protein was analyzed in RNF141 64 pairs of CRC and adjacent normal tissues. (E) The effect of RNF141 overexpression on KRAS membrane/cytoplasmic localization was visualized by IF assay. Scale bar, 20 μm.

**Fig. s6 Overexpression of RNF141 upregulates MEK-ERK signaling, promotes proliferation and inhibits apoptosis.** (A) The effects of RNF141 overexpression on protein levels of MEK, p-MEK, ERK, p-ERK was assessed by Western blot. (B and C) Protein levels of PCNA, Cleaved caspase-3, Cleaved PARP, KRAS and RNF141 in SW480 (B) and DLD-1 (C) cells co-transfected with indicated siRNA and lentivirus were validated by western blot. Data were mean ± SD from three independent experiments, each performed in triplicate. ** *P* < 0.01, *** *P* < 0.001.
