# Supplementary material for: RNF141 interacts with KRAS to promote colorectal cancer progression
Source: Oncogene. 2021 Aug 3;40(39):5829–42. doi: 10.1038/s41388-021-01877-4 (PMC8484013; doi:10.1038/s41388-021-01877-4)
Supplement: Supplementary file 3 — Supplementary table 1 [file 41388_2021_1877_MOESM3_ESM.docx]

| KRAS exon | KRAS mutations | n (%) |
| --- | --- | --- |
| Exon 2 | p.G12D | 6 |
|  | p.G12V | 3 |
|  | p.G12A | 2 |
|  | p.G12C | 1 |
|  | p.G12S | 1 |
|  | p.G12R | 1 |
|  | p.G13D | 5 |
| Exon 3 | p.A59T | 1 |
|  | p.Q61H | 1 |
| Exon 4 | p.A146T | 1 |
|  | p.A146V | 1 |
|  | Others | 1 |
| Total |  | 24 (37.5%) |

Supplementary table 1

Incidence of KRAS mutation [n (%)]
