# Supplementary figures and images for: RNF141 interacts with KRAS to promote colorectal cancer progression
Source: Oncogene. 2021 Aug 3;40(39):5829–42. doi: 10.1038/s41388-021-01877-4 (PMC8484013; doi:10.1038/s41388-021-01877-4)

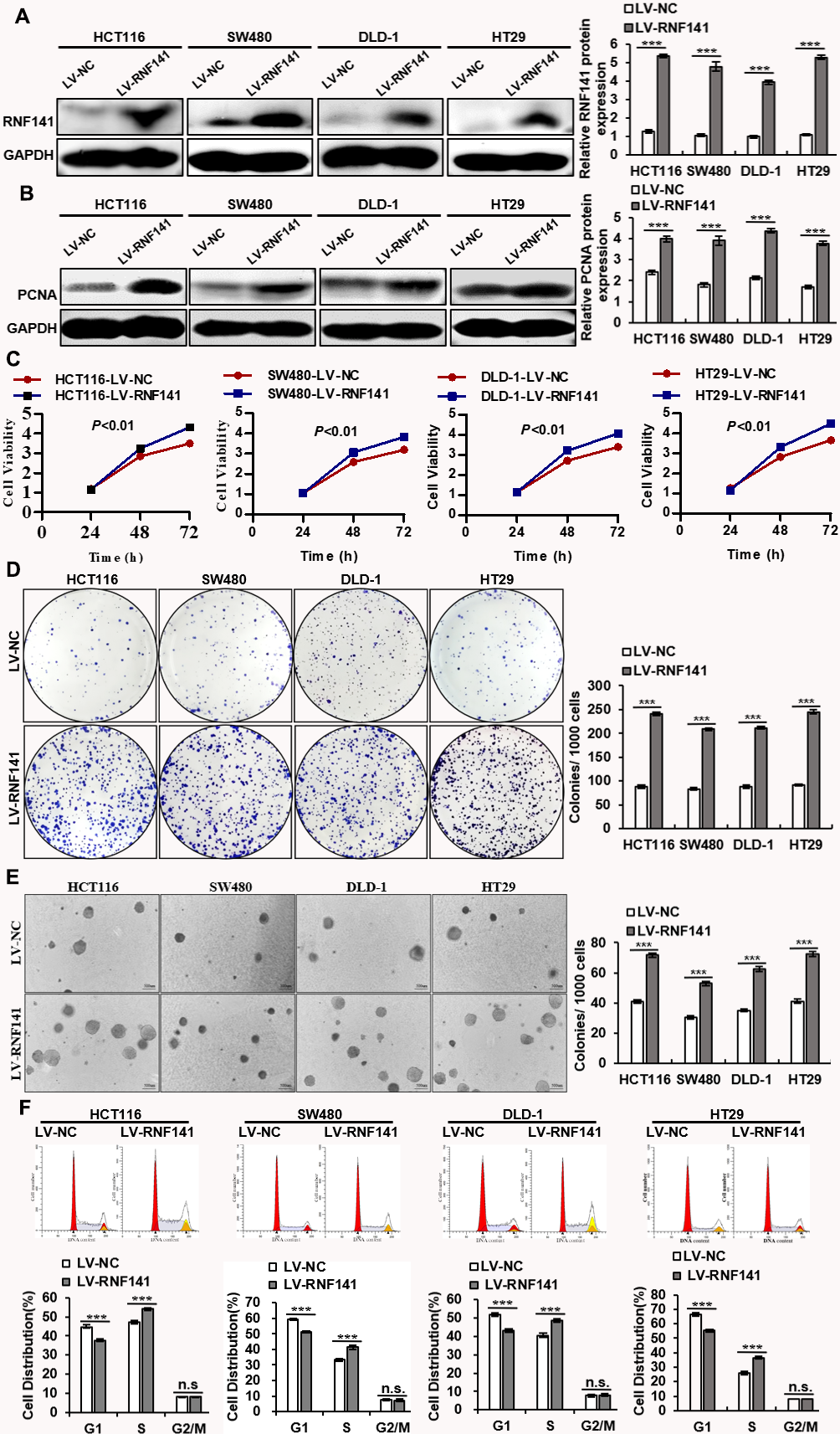

Supplement: Supplementary file 4 — Fig. s1 [file 41388_2021_1877_MOESM4_ESM.tif]

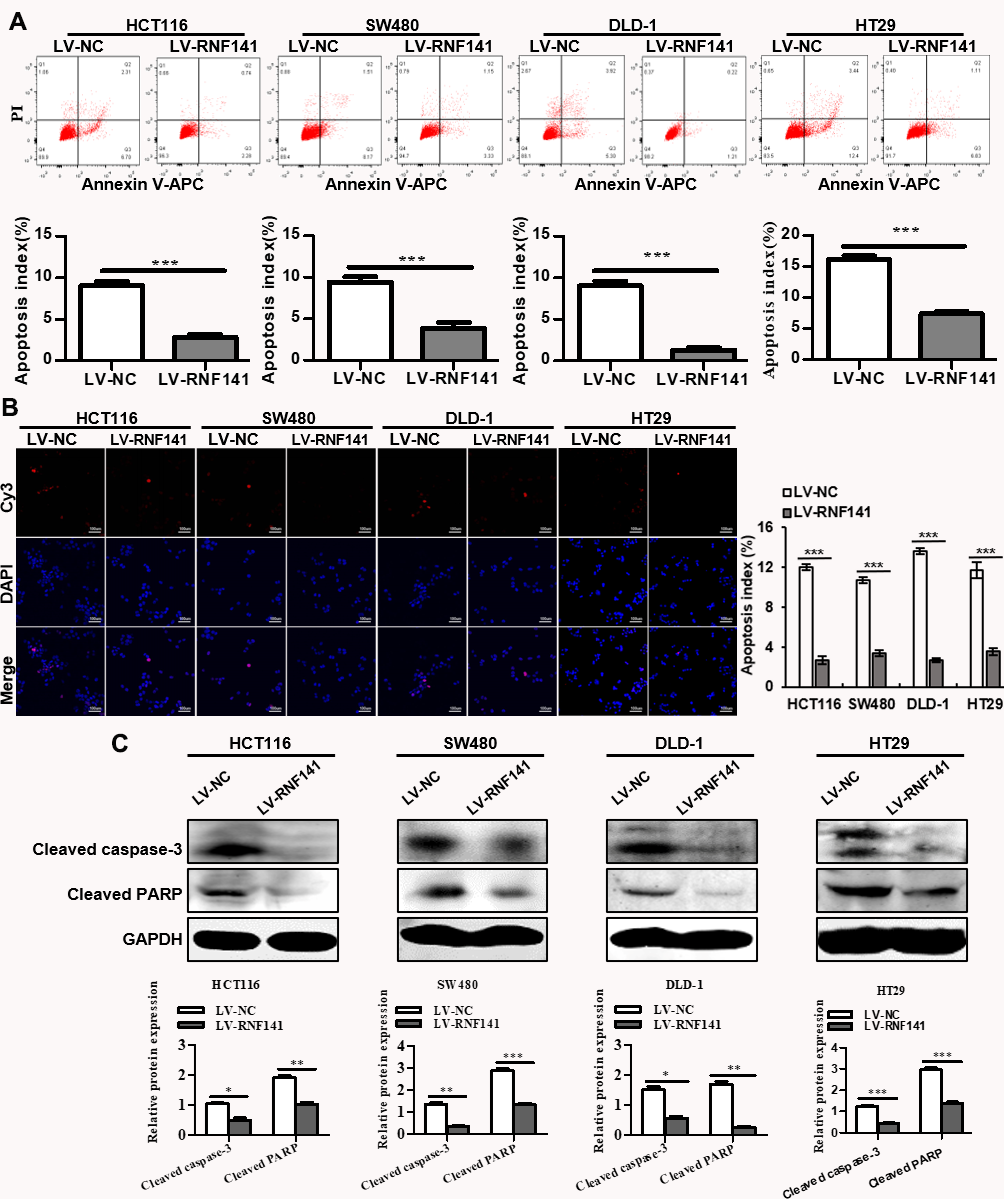

Supplement: Supplementary file 5 — Fig. s2 [file 41388_2021_1877_MOESM5_ESM.tif]

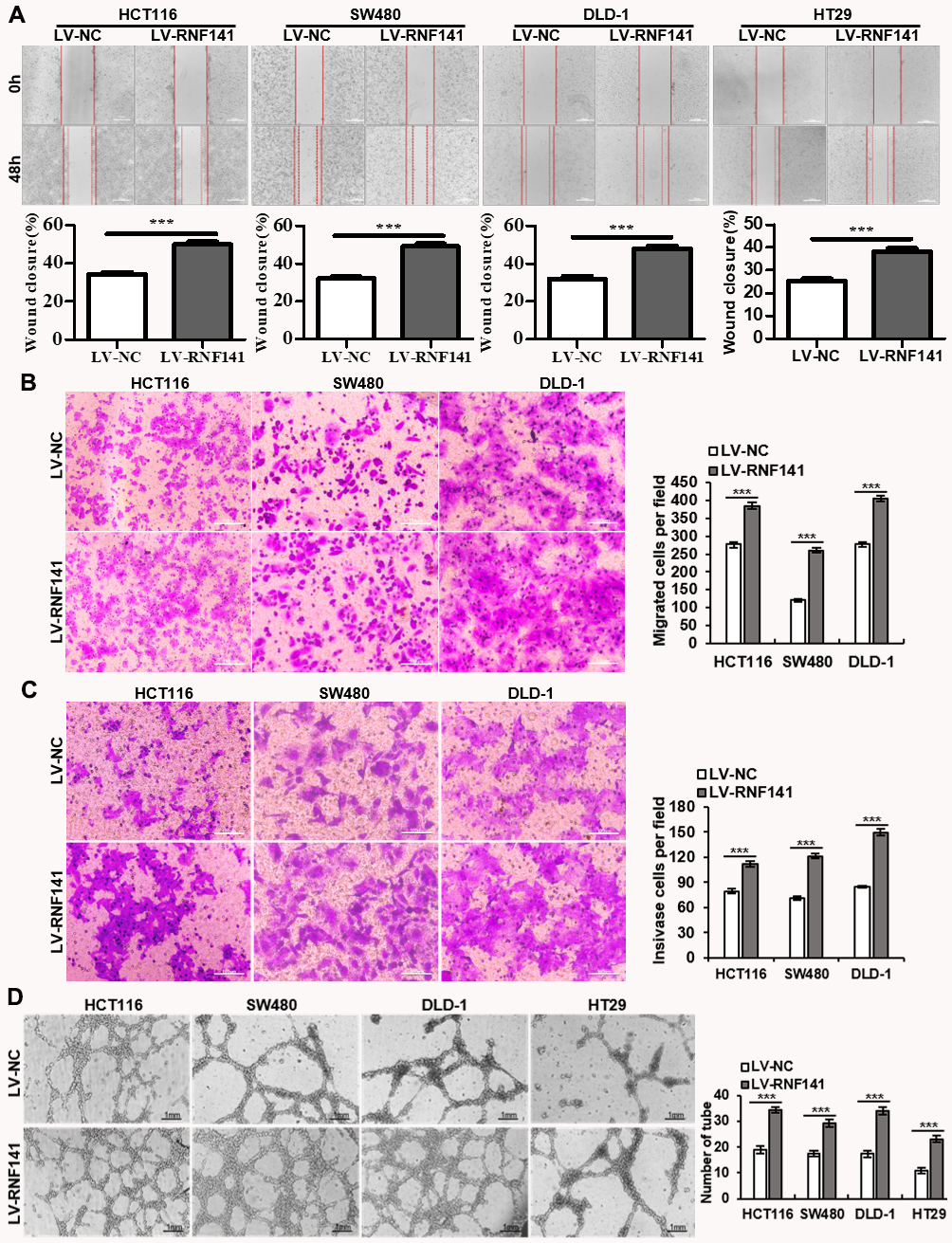

Supplement: Supplementary file 6 — Fig. s3 [file 41388_2021_1877_MOESM6_ESM.tif]

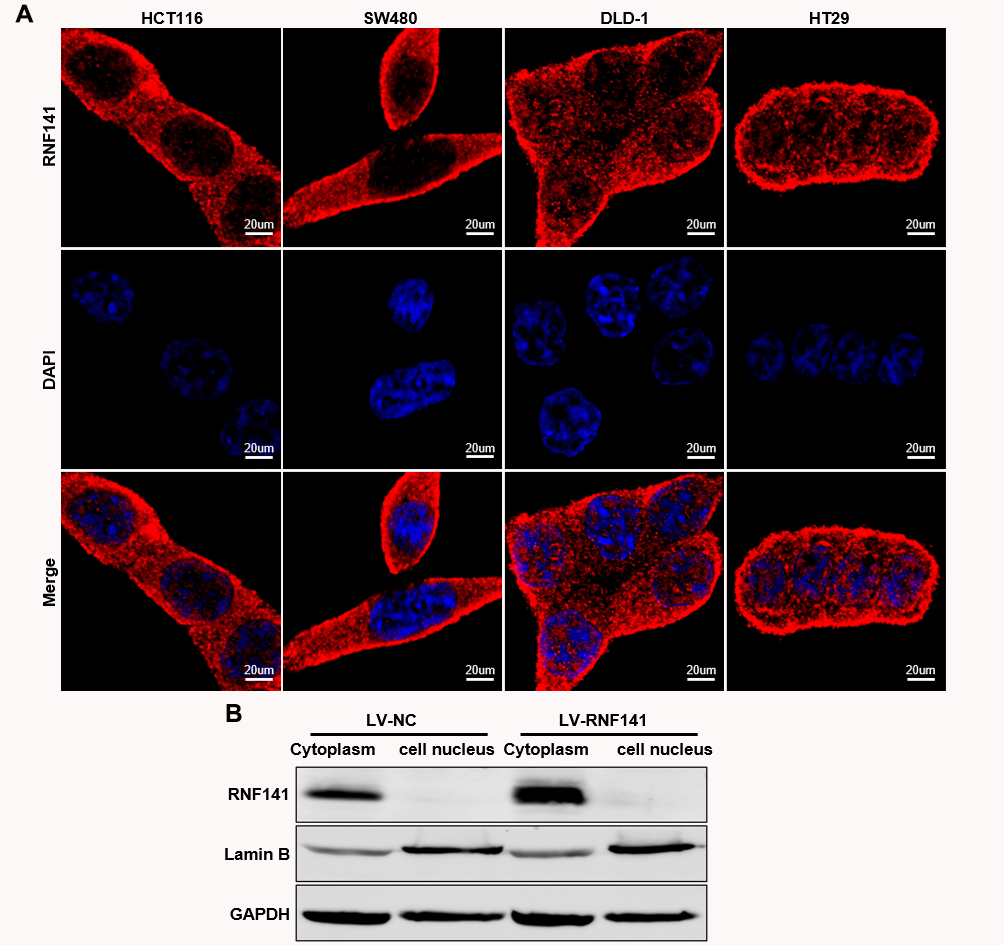

Supplement: Supplementary file 7 — Fig. s4 [file 41388_2021_1877_MOESM7_ESM.tif]

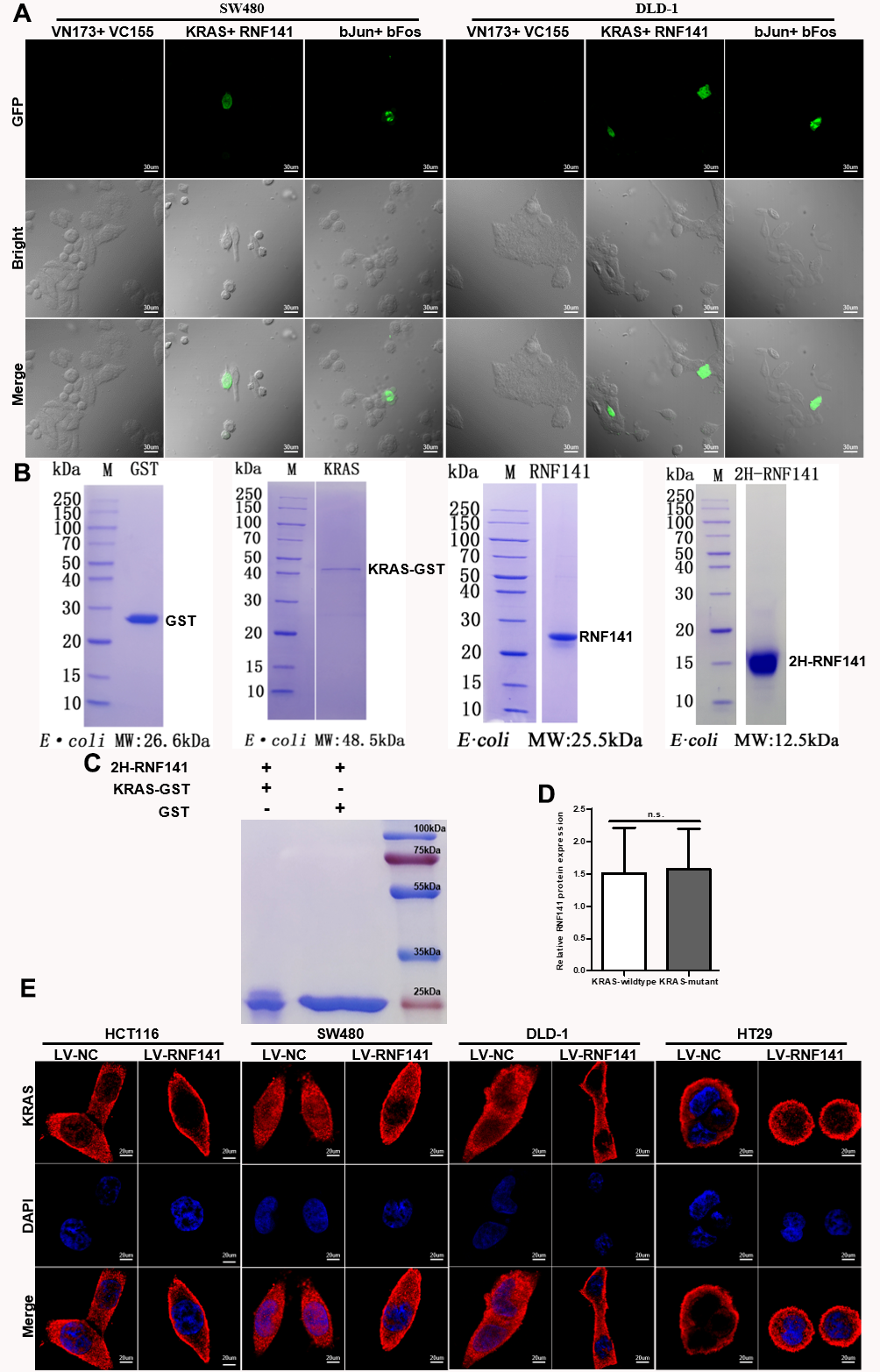

Supplement: Supplementary file 8 — Fig. s5 [file 41388_2021_1877_MOESM8_ESM.tif]

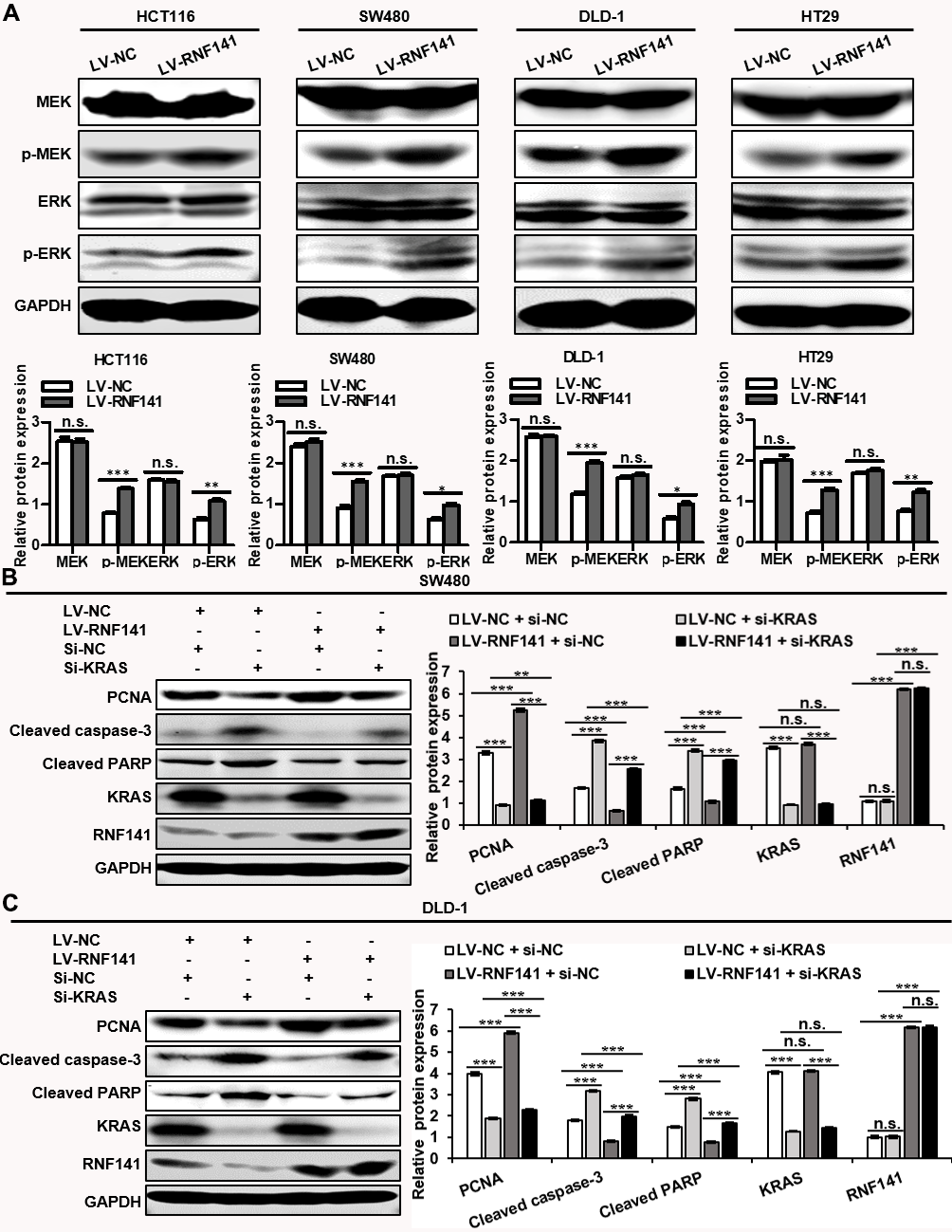

Supplement: Supplementary file 9 — Fig. s6 [file 41388_2021_1877_MOESM9_ESM.tif]
