# Supplementary material for: RNF141 interacts with KRAS to promote colorectal cancer progression
Source: Oncogene. 2021 Aug 3;40(39):5829–42. doi: 10.1038/s41388-021-01877-4 (PMC8484013; doi:10.1038/s41388-021-01877-4)
Supplement: Supplementary file 12 — Supplementary data 3 [file 41388_2021_1877_MOESM12_ESM.pdf]

动物实验福利与伦理审查决议
Approval Letter of Laboratory Animal Welfare and Ethical

项目名称<br>**Study Title**	RNF141 通过与 KRAS 相互作用促进结直肠癌的发生发展		
项目来源<br>**Issued BY**	自选项目		
主要研究者<br>**Principal investigator**	姜慧卿	科室<br>**Department**	消化内科
送审日期<br>**Date Submitted**	2021.3.8	审查决议编号<br>**Approval Letter No.**	2021-AE016
动物来源<br>**Animal origin**	采购（Procurement）自北京维通利华实验动物技术有限公司		
品种/品系<br>**breed/strain**	裸鼠		
动物级别<br>**Grade**	SPF级	数量（只）<br>**Number (♀；♂)**	雌（♀）________；<br>雄（♂）20 ____；
周/月龄<br>**W/M Age**	4周	体重（g）<br>**Weight**	14-16g
计划执行时间<br>**Period of Protocol**	2021 年 1 月 1 日 至 2021 年 6 月 30 日		
审查意见和结论<br>**Review opinions and conclusions**	根据该研究的实验设计，经伦理委员会审查，符合动物保护、动物福利和伦理原则，符合国家实验动物福利伦理的相关规定。<br>According to the experimental design of the study, after the review of the ethics committee, that it was in line with the principles of animal protection, animal welfare and ethics, and the relevant provisions of the national experimental animal welfare ethics.<br>主审委员（签字）________<br>主任委员（签字）________<br>日期：2021 年 4 月 9 日<br>河北医科大学第二医院科研伦理委员会		

地址：河北省石家庄市新华区和平西路 215 号　　邮编：050051　　电话/传真：0311-66002811
Address: No.215 Heping West Road, Xinhua District, Shijiazhuang ,Hebei, 050051,Tel/Fax:0311-66002811
