# Supplementary material for: RNF141 interacts with KRAS to promote colorectal cancer progression
Source: Oncogene. 2021 Aug 3;40(39):5829–42. doi: 10.1038/s41388-021-01877-4 (PMC8484013; doi:10.1038/s41388-021-01877-4)
Supplement: Supplementary file 13 — Supplementary data 4 [file 41388_2021_1877_MOESM13_ESM.pdf]

科研伦理委员会伦理审查决议
Approval Letter of Research Ethics Committee

项目名称<br>**Study Title**	RNF141 通过与 KRAS 相互作用促进结直肠癌的发生发展		
项目来源<br>**Issued BY**	自选项目		
主要研究者<br>**Principal investigator**	姜慧卿	科室<br>**Department**	消化内科
审查类别<br>**Category of Review**	初始审查	审查方式<br>**Type of Review**	快速审查
送审日期<br>**Date Submitted**	2021-03-02	审查决议编号<br>**Approval Letter No.**	2021-P014
会议地点<br>**Meeting Location**		会议时间<br>**Meeting Date**	
送审资料<br>**Document(s) Reviewed**	研究方案（版本号 1.0，版本日期：2021.03.01）<br>知情同意书（版本号 1.0，版本日期：2021.03.01）<br>主要研究者简历		
投票结果<br>**Comments**	伦理委员会按照国家相关法规要求对送审材料进行审阅和讨论，出席会议_0_人，参与投票（Vote）_0_人。<br>投票结果：同意（Approved）_0_票；作必要的修正后同意（Conditional approved）_0_票；作必要修正后重审（Reviewd after revising）_0_票；终止或暂停已批准的研究（Termination or suspension of approved studies）_0_票；不同意（Rejected）_0_票。		
审查意见和结论<br>**Review opinions and conclusions**	根据国家卫健委《涉及人的生命科学和医学研究伦理审查办法》、《赫尔辛基宣言》和《涉及人的健康相关研究国际伦理指南》的伦理原则，经本伦理委员会审查，同意（Approved）按所批准的临床研究方案、知情同意书开展本项研究。<br><br>主审委员（签字）[signature]<br>主任委员（签字）[stamp: 崔炜]<br>日期：2021 年 3 月 8 日<br>[stamp: 河北医科大学第二医院 科研伦理委员会]		

地址：河北省石家庄市新华区和平西路 215 号　　邮编：050051　　电话/传真：0311-66002811
Address: No.215 Heping West Road, Xinhua District, Shijiazhuang ,Hebei, 050051,Tel/Fax:0311-66002811

请遵循 GCP 原则、遵循伦理委员会批准的方案开展临床研究，保护受试者的健康与权利。研究过程中若变更主要研究者，对临床研究方案、知情同意书、招募材料等的任何修改，请申请人提交修正案审查申请。发生严重不良事件，及时提交严重不良事件报告。发生违反试验方案情况须及时报告本伦理委员会。

In order to protect the health and rights of the subjects, the applicant should follow the GCP principles and programs approved by the Ethical Committee during the clinical studies. If any changes are made to the primary investigator(s), the clinical research protocol, informed consent, recruitment materials, etc., the applicant is requested to submit an amendment for review. In the event of a serious adverse event, a serious adverse event report should be submitted in time. The violation of the protocol shall be promptly reported to the Ethics Committee.

请按照伦理委员会规定的年度/定期跟踪审查频率，在截止日期前 1 个月提交研究进展报告；申请人暂停或提前终止临床研究，请及时提交暂停/终止研究报告。完成临床研究，请申请人提交结题报告。

The applicant should submit the study progress report one month before the deadline according to the frequency of the annual / periodic follow-up examination. If the applicant suspends or prematurely terminates the clinical research, the suspension / termination report should be submitted in time. The applicant should submit the final report when they complete the clinical study.

研究纳入了不符合纳入标准或符合排除标准的受试者，符合中止试验规定而未让受试者退出研究，给予错误治疗或剂量，给予方案禁止的合并用药等没有遵从方案开展研究的情况；或可能对受试者的权益/健康以及研究的科学性造成不良影响等违背 GCP 原则的情况，请申办者/监察员/研究者提交违背方案报告。

If the study is not carried out in accordance with the scheme,such as the study included subjects who did not meet the inclusion criteria or met the exclusion criteria, subjects were not withdrawn from the study in accordance with the termination rule, the wrong treatment or dosage was given, the combination drugs prohibited by the scheme were given, or the rights / health of the subjects and the scientific nature of the study may be adversely affected, which violated the GCP principle, the sponsor / inspector / researcher is requested to submit the violation report.

本试验年度/定期跟踪审查频率为一年，本批件有效期为一年。

The annual / periodic follow-up examination frequency is one year and the validity of this document is one year.

地址：河北省石家庄市新华区和平西路 215 号　　邮编：050051　　电话/传真：0311-66002811
Address: No.215 Heping West Road, Xinhua District, Shijiazhuang ,Hebei, 050051,Tel/Fax:0311-66002811
